# Dissecting the effect of mitochondrial BCAT inhibition in methylmalonic acidemia

**DOI:** 10.1172/jci.insight.187758

**Published:** 2025-09-09

**Authors:** Madeline G. Hemmingsen, Guo-Fang Zhang, Yunhan Ma, Hannah Marchuk, Kalyani R. Patel, Tong Chen, Xinning Li, Mark Chapman, Sabrina Collias, Dolores H. Lopez-Terrada, James Beasley, Ashlee R. Stiles, Randy J. Chandler, Charles P. Venditti, Sarah P. Young, Mercedes Barzi, Beatrice Bissig-Choisat, Doug Krafte, Christopher B. Newgard, Karl-Dimiter Bissig

**Affiliations:** 1 Alice and Y. T. Chen Center for Genetics and Genomics, Division of Medical Genetics, Department of Pediatrics;; 2 University Program in Genetics and Genomics;; 3 Duke Molecular Physiology Institute and Sarah W. Stedman Nutrition and Metabolism Center; and; 4 Department of Medicine, Division of Endocrinology, Metabolism, and Nutrition, Duke University School of Medicine, Durham, North Carolina, USA.; 5 Department of Biomedical Engineering, Duke University Pratt School of Engineering, Durham, North Carolina, USA.; 6 Department of Pathology and Immunology, Baylor College of Medicine and Texas Children’s Hospital, Houston, Texas, USA.; 7 Department of Molecular Genetics and Microbiology, Duke University Medical Center, Durham, North Carolina, USA.; 8 OmniAb Inc., Durham, North Carolina, USA.; 9 Department of Pediatrics, Baylor College of Medicine and Texas Children’s Hospital, Houston, Texas, USA.; 10 Duke University Health System Biochemical Genetics Laboratory, Durham, North Carolina, USA.; 11 National Human Genome Research Institute, National Institutes of Health (NIH), Bethesda, Maryland, USA.; 12 Department of Pharmacology and Cancer Biology;; 13 Department of Medicine, Division of Gastroenterology; and; 14 Duke Cancer Center, Duke University Medical Center, Durham, North Carolina, USA.; 15 Duke Regeneration Center, Duke University School of Medicine, Durham, North Carolina, USA.; 16 Center for Advanced Genomic Technologies, Duke University, Durham, North Carolina, USA.

**Keywords:** Genetics, Metabolism, Amino acid metabolism

## Abstract

Methylmalonic acidemia (MMA) is a severe metabolic disorder affecting multiple organs because of a distal block in branched-chain amino acid (BCAA) catabolism. Standard of care is limited to protein restriction and supportive care during metabolic decompensation. Severe cases require liver/kidney transplantation, and there is a clear need for better therapy. Here, we investigated the effects of a small molecule branched-chain amino acid transaminase (BCAT) inhibitor in human MMA hepatocytes and an MMA mouse model. Mitochondrial BCAT is the first step in BCAA catabolism, and reduction of flux through an early enzymatic step is successfully used in other amino acid metabolic disorders. Metabolic flux analyses confirmed robust BCAT inhibition, with reduction of labeling of proximal and distal BCAA-derived metabolites in MMA hepatocytes. In vivo experiments verified the BCAT inhibition, but total levels of distal BCAA catabolite disease markers and clinical symptoms were not normalized, indicating contributions of substrates other than BCAA to these distal metabolite pools. Our study demonstrates the importance of understanding the underlying pathology of metabolic disorders for identification of therapeutic targets and the use of multiple, complementary models to evaluate them.

## Abstract 2

Many amino acid disorders arise from a downstream block in their degradation pathways, leading to the accumulation of toxic intermediates. Here, we used metabolic flux analysis to evaluate the inhibition of branched-chain amino acid (BCAA) transaminase (BCAT), a proximal step in the catabolic pathway, as a therapeutic approach for methylmalonic acidemia. This strategy, e.g. the reduction of flux through the defective pathway, was successfully used in other amino acid disorders. Surprisingly, BCAT inhibition failed to ameliorate accumulation of toxic distal catabolites, despite the robust inhibition of the proximal BCAA catabolism. Our findings underscore the necessity of understanding the underlying pathology of metabolic disorders to identify therapeutic targets and highlight the value of using diverse, complementary models for evaluating treatments.

## Graphical abstract

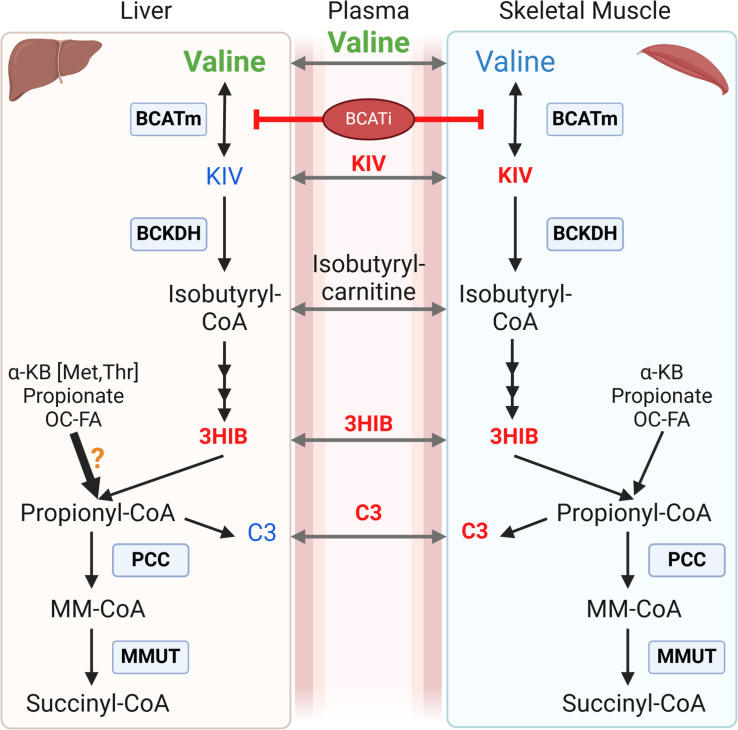

## Introduction

Methylmalonic acidemia (MMA) is an organic aciduria resulting from a defect in the isomerization of methylmalonyl-CoA into succinyl-CoA during valine and isoleucine catabolism. MMA is a genetically heterogeneous disorder, with the majority (~60%) of cases caused by complete (*mut^0^*) or partial (*mut*^–^) deficiency of the enzyme methylmalonyl-CoA mutase (MMUT) and the remainder due to errors in the synthesis or transport of 5′-deoxyadenosylcobalamin, a vitamin B_12_ derivative and essential cofactor of the reaction (1, 2). A block in MMUT activity leads to accumulation of methylmalonic acid and other propionyl-CoA derived catabolites, which in turn have secondary effects on associated pathways and play a major role in the pathogenesis of MMA (3). The disorder affects 1 in 50,000 to 100,000 individuals (4–6) and has a wide multisystemic spectrum of symptoms. The major clinical features of MMA include propensity for ketoacidosis, lethargy, failure to thrive, vomiting, and muscular hypotonia (7, 8), with long-term complications, such as renal tubular dysfunction, intellectual disability, and hepatomegaly (7, 9–11). There is currently no targeted therapy approved for MMA, and treatment is focused on decreasing metabolic stress through dietary protein restriction, l-carnitine supplementation, and cofactor supplementation (8, 10, 12). Even with strict management of diet and care, mortality is still substantial (6%–20%), with high risk of metabolic decompensation and disease-associated sequelae (12, 13). Elective liver and combined liver-kidney transplantation have become the last therapeutic resort for more severely affected patients, conferring a measure of metabolic stability and allowing a less stringent diet (14–18). Nevertheless, the finite number of liver donors, persistence of disease symptoms, risk of transplant-related complications, and lifelong immune suppression limit the utility of this approach for MMA. More effective and less invasive therapies are clearly needed.

**Figure 1:**
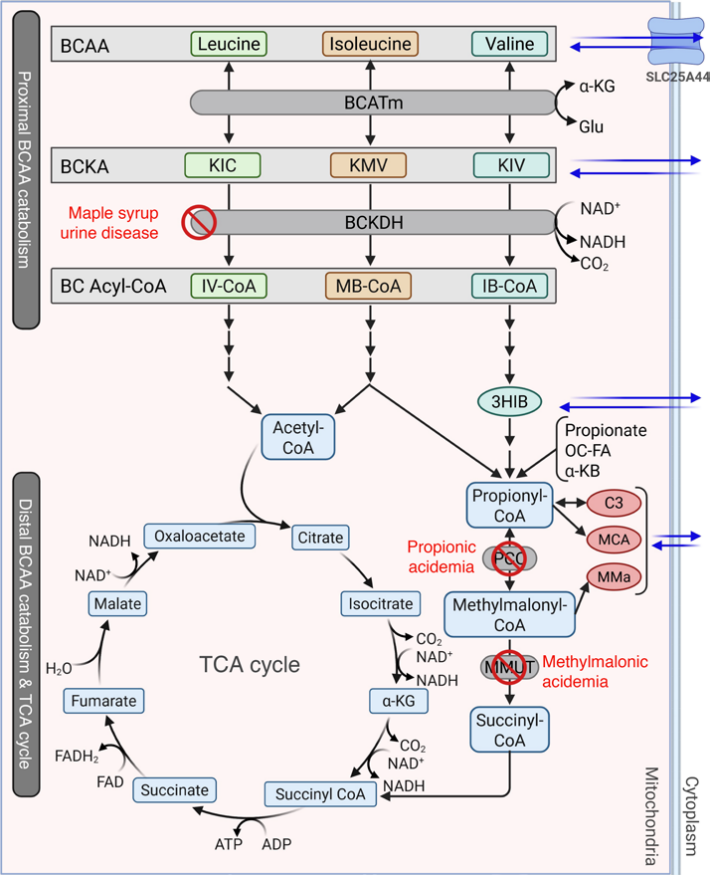
Branched-chain amino acid metabolism. Diagram of the mitochondrial branched-chain amino acid (BCAA) catabolic pathway. BCAAs are transported into the mitochondria through SLC25A44, where mitochondrial BCAT (BCATm) catalyzes reversible transamination of BCAAs to produce the branched-chain ketoacids (BCKAs) α-ketoisovalerate (KIV), α-keto-α-methylvalerate (KMV), and α-ketoisocaproate (KIC). BCKAs can be irreversibly oxidized by branched-chain ketoacid dehydrogenase (BCKDH) to form branched-chain acyl-CoA (BC Acyl-CoA) thioesters isovaleryl-CoA (IV-CoA), methylbutyryl-CoA (MB-CoA), and isobutyryl-CoA (IB-CoA), which are retained in the mitochondria for further oxidation to enter the tricarboxylic acid (TCA) cycle as either acetyl-CoA or succinyl-CoA. Succinyl-CoA is also generated as a product of propionyl-CoA metabolism, a substrate formed from the oxidative metabolism of isoleucine and valine, from propionate from gut flora, from odd-chain fatty acid (OC-FA) metabolism, and from metabolism of methionine and threonine in the form of α-ketobutyrate (α-KB). In methylmalonic acidemia, propionylcarnitine (C3), 2-methylcitrate (MCA), and methylmalonic acid (MMA) are accumulated during propionyl-CoA metabolism. 3HIB, 3-hydroxyisobutyrate.

Inhibition of enzymes upstream of a block in a metabolic pathway has long been used as a successful therapeutic approach for monogenic liver diseases, such as hereditary tyrosinemia (19). We have also previously shown that metabolic pathway reprogramming through CRISPR-mediated knockout of upstream enzymes successfully rescues the disease phenotype in models of both tyrosinemia and glutaric aciduria (20, 21). Therefore, we aimed to explore the therapeutic potential of inhibition of an enzyme proximal to MMUT in the branched-chain amino acid (BCAA) degradation pathway, which leads to MMA. The first step of BCAA catabolism is a rapid and reversible transamination of leucine, isoleucine, and valine by branched-chain amino acid transaminase (BCAT) to form glutamate and the corresponding branched-chain α-ketoacids (BCKAs) (Figure 1). Cytosolic (BCATc) and mitochondrial (BCATm) forms of BCAT are encoded by *BCAT1* and *BCAT2*, respectively (22). In addition to localizing to distinct subcellular compartments, the 2 isozymes of BCAT segregate in specific tissues, with BCATc found only in the central nervous system (23, 24) and BCATm expressed in most tissues. The highest BCATm activity in rodents is observed in stomach, heart, kidney, brain, and skeletal muscle (25), while BCATm is expressed at very low levels in the liver (24, 26). Whole-body knockout of *Bcat2* yields healthy and viable mice, despite elevated plasma BCAAs (27). However, little research has been done toward investigating BCATm as a therapeutic target outside of its possible use for obesity, dyslipidemia, and some forms of cancer (28–30). The impact of modulation of BCATm in a disease state such as MMA is unknown.

MMA mouse models have been instrumental tools for the preclinical evaluation of new therapeutics (31, 32). Full *Mmut*-knockout mice (*Mmut^–/–^*) display neonatal lethality (33, 34), and while useful for gene therapy studies (35–38), the severe phenotype limits physiological studies that require adult animals. Tissue-specific transgene models have allowed for the dissection of renal and hepatic pathophysiology (11, 39–41) while knockins of selected patient mutations have yielded either a mild hypomorphic phenotype or substantial preweaning lethality (42, 43). Although previous MMA mouse models recapitulate human disease, a model of MMA that has prolonged adult survival with the replication of the metabolic and clinical phenotypes seen in severe patients has not been developed.

Here we evaluate the effect of a BCATm inhibition on BCAA catabolism using 2 different yet complementary model systems. First, we use human hepatocytes from healthy donors and patients with MMA studied in vitro by measurement of metabolism of stable isotope-labeled BCAA and BCKA to determine the direct impact of BCATm inhibition in a relevant human and disease setting. Our results demonstrate efficient inhibition of BCATm in healthy and MMA hepatocytes and clearly define abnormalities in metabolic flux caused by mutation of *MMUT*. Second, to study the systemic impact of BCAT inhibition, we developed a new MMA mouse model that mimics severe human disease phenotypes and allows studies in adult animals. Valine flux analysis in vivo verified the inhibition of BCATm but also demonstrated that this strategy is unable to lower distal BCAA catabolites and disease biomarkers, likely due to influx of metabolic substrates distal to the BCATm block.

## Results

### Inhibition of BCATm in patient-derived MMA hepatocytes.

**Figure 2:**
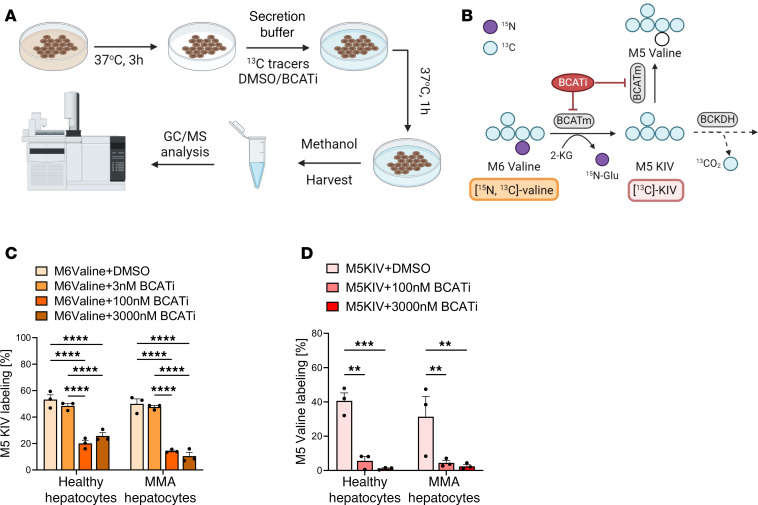
BCAT inhibition in healthy and patient-derived MMA hepatocytes in primary culture. (**A**) Schematic of experimental protocol in cultured control (healthy) and MMA hepatocytes. (**B**) Schematic of isotope tracing with labeled [^15^N, ^13^C_5_]valine or [^13^C_5_]KIV. [^15^N, ^13^C_5_]valine can be transaminated to M5 KIV, and [^13^C_5_]KIV can be reaminated to M5 valine or oxidized by BCKDH. (**C**) Quantification of M5 KIV labeling in healthy and MMA hepatocytes supplemented with 800 μM [^15^N, ^13^C_5_]valine. (**D**) Quantification of M5 valine labeling in healthy and MMA hepatocytes supplemented with 800 μM [^13^C_5_]KIV. All results are mean ± SEM and analyzed by 1- or 2-way ANOVA. “**” *P* ≤ 0.01, “***” *P* ≤ 0.001, “****” *P* ≤ 0.0001. GC/MS, gas chromatography–mass spectrometry.

To identify a potent and specific BCATm inhibitor, we performed a structure-based medicinal chemistry optimization starting from a published lead compound (compound 8b) (44), leveraging the cocrystal structure available in the US data center for the global Protein Data Bank (https://www.rcsb.org, code: 5HNE). Over 180 analogs were synthesized and tested in BCATm and BCATc enzymatic assays. Lead compounds were profiled against other receptors and a kinase panel and found to be selective for BCATm inhibition. We identified a potent (IC_50_ = 17 nM) and specific BCATm inhibitor (BCATi) with very good selectivity for BCATm over BCATc (IC_50_ = 508 nM) (Supplemental Figure 1; supplemental material available online with this article; https://doi.org/10.1172/jci.insight.187758DS1). To evaluate its impact on human cells, we performed metabolic flux analyses with labeled [^15^N, ^13^C_5_]valine or [^13^C_5_]α-ketoisovalerate ([^13^C_5_]KIV) in plated hepatocytes derived from a healthy donor or from patients with MMA who received a liver transplant (Figure 2A). The 2 primary readouts of BCATm activity are M5 KIV, formed by transamination of [^15^N, ^13^C_5_]valine, and M5 valine, produced by reverse transamination of [^13^C_5_]KIV (Figure 2B). When provided [^15^N, ^13^C_5_]valine, both healthy and MMA hepatocytes treated with doses of BCATi of 100 nM or higher had M5 KIV pools with lower percentage labeling compared with treatment with DMSO as control (Figure 2C). Labeling of M5 valine in both healthy and MMA hepatocytes provided with [^13^C_5_]KIV as substrate was also strongly impaired by BCATi at doses of at least 100 nM, consistent with effective inhibition of the reverse transaminase function of BCATm (Figure 2D).

**Figure 3:**
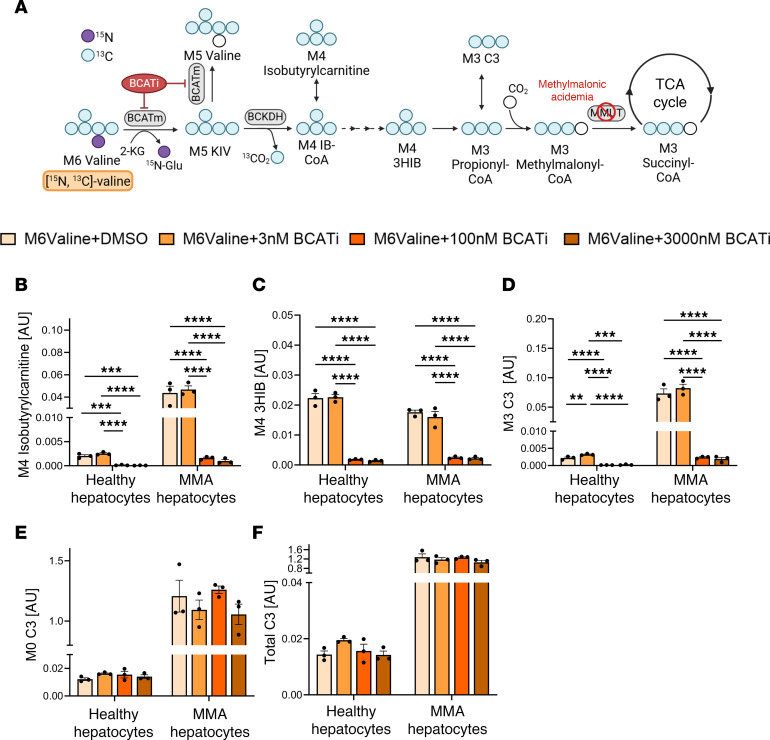
Evaluation of BCATi in isolated hepatocyte cultures. (**A**) Schematic of isotope tracing with labeled [^15^N, ^13^C_5_]valine. (**B**–**F**) Quantification of M4 isobutyrylcarnitine (**B**), M4 3HIB (**C**), M3 C3 acylcarnitine (**D**), M0 C3 acylcarnitine (**E**), and total C3 acylcarnitine (**F**) in healthy and MMA hepatocytes supplemented with 800 μM [^15^N, ^13^C_5_]valine. Data expressed as arbitrary units representing peak areas for a given metabolite relative to peak area of an added norvaline standard for 3HIB and [^2^H_9_]l-carnitine (M9 carnitine) internal standard for M4 isobutyrylcarnitine and C3 acylcarnitine. All results are mean ± SEM and analyzed by 1- or 2-way ANOVA, with statistical significance indicated by the symbols “**” *P* ≤ 0.01, “***” *P* ≤ 0.001, “****” *P* ≤ 0.0001.

To evaluate the inhibitory effect of BCATi on products produced in later stages of the valine catabolic pathway, we measured labeling of M4 isobutyrylcarnitine, M4 3HIB, and M3 propionylcarnitine (M3 C3) (see Figure 3A and schematic, Figure 1). In healthy and MMA hepatocytes supplemented with [^15^N, ^13^C_5_]valine, BCATi at ≥100 nM caused a significant decrease in the concentrations of M4 isobutyrylcarnitine (Figure 3B), M4 3HIB (Figure 3C), and M3 C3 (Figure 3D). The concentration of each of these labeled metabolites was measured against norvaline as an internal standard. In cells incubated with [^13^C_5_]KIV, we observed increases in the concentrations of M4 isobutyrylcarnitine and M4 3HIB as the dose of BCATi increased, consistent with a shift toward higher use of the labeled substrate due to inhibition of reverse transamination (Supplemental Figure 2, B and C). M4 isobutyrylcarnitine is produced from M4 IB-CoA, a product of the second step of BCAA catabolism, whereas 3HIB is generated from M4 IB-CoA and metabolized to propionyl-CoA, which rapidly equilibrates to form C3 (Figure 1). Importantly, despite the dramatic decrease in the level of labeled M3 C3 in healthy and MMA hepatocytes treated with BCATi (Figure 3D), treatment with the inhibitor had no significant effect on the concentration of the unlabeled pool of C3 (M0 C3, Figure 3E) or the concentration of the total pool of C3 (M3 C3 + M0 C3 = total C3, Figure 3F) in healthy or MMA hepatocytes exposed to [^15^N, ^13^C_5_]valine. As expected, due to their deficiency in MMUT, MMA hepatocytes had higher concentrations of M3 C3, M0 C3, and total C3 than healthy hepatocytes (Figure 3, D–F). These findings suggest that valine (or BCAA more generally) is a relatively minor contributor to the pool of C3 in normal or MMA hepatocytes, given the lack of effect of the BCATi on M0 C3 or total C3. This lack of effect on the M0 or total pools of C3 occurs despite the clear inhibition of formation of the labeled valine products M4 isobutyrylcarnitine, M4 3HIB, and M3 C3 by treatment with the BCATi. Taken together, these data are consistent with effective inhibition of BCAT by the BCATi but minimal effect on the M0 or total C3 pools due to low BCAT activity in hepatocytes.

**Figure 4:**
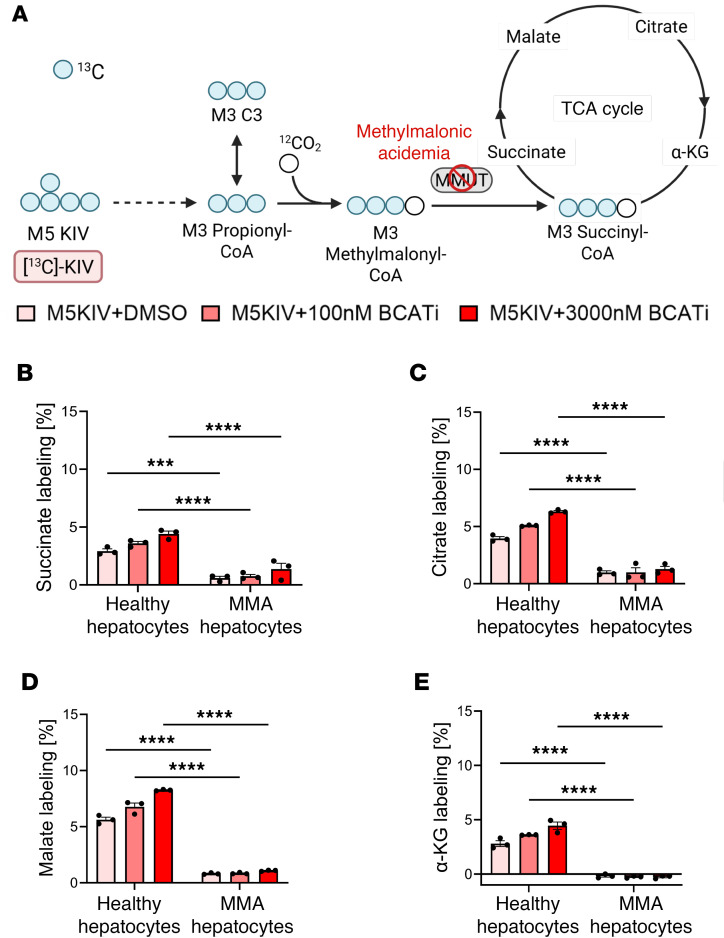
Flux of KIV into the TCA cycle. (**A**) Schematic of isotope tracing with labeled [^13^C_5_]KIV into the TCA cycle. (**B**–**E**) Quantification of average percentage carbon labeling of TCA intermediates succinate (**B**), citrate (**C**), malate (**D**), and α-ketoglutarate (α-KG) (**E**) in control and MMA hepatocytes supplemented with 800 μM [^13^C_5_]KIV. Results are reported as mean ± SEM and analyzed by 2-way ANOVA, with statistical significance indicated by the symbols “***” *P* ≤ 0.001, “****” *P* ≤ 0.0001.

To assess downstream flux of valine or KIV into the TCA cycle, we measured enrichment of labeled succinate, malate, citrate, and α-ketoglutarate (α-KG) in the healthy and MMA hepatocytes incubated with labeled valine or KIV as substrates, with and without a range of BCATi concentrations (Figure 4A). TCA cycle intermediates were undetectable when [^15^N, ^13^C_5_]valine was used as substrate, likely reflecting the low intrinsic levels of BCAT in the human liver and the further decrease in expression of metabolic genes (including BCAT) known to occur upon plating of hepatocytes. When [^13^C_5_]KIV was used as substrate, the TCA cycle intermediates were now clearly labeled, but MMA hepatocytes had significantly lower incorporation of ^13^C into these intermediates under all experimental conditions compared with healthy hepatocytes (Figure 4, B–E). This reflects the block of methylmalonyl-CoA conversion to succinyl-CoA in MMA. As expected, the BCATi had no significant effect on labeling of any of the 4 TCA cycle intermediates when [^13^C_5_]KIV was used as substrate.

In sum, the data presented for isolated MMA and normal human hepatocytes demonstrate a clear effect of the BCATi to reduce labeling of products derived from valine catabolism, as well as an inhibitory effect on the reverse BCATm reaction that converts KIV to valine. However, this robust inhibition did not affect the concentration of M0 C3 or total C3 acylcarnitine, suggesting that the contribution of valine catabolism to the pool of C3 acylcarnitine is limited by the low expression level of BCAT in plated human hepatocytes and that other substrates make key contributions to these distal pools of intermediates.

### Generation of the Mmut^p.L690Ins/p.L690Ins^ mouse strain.

Although our findings with normal and MMA human hepatocytes clearly demonstrate a lack of effect of BCAT inhibition on the levels of distal BCAA metabolites that accumulate with MMUT deficiency, a different outcome remains possible with use of a BCATi in the in vivo setting. This is because administration of a BCATi in vivo would be expected to inhibit the target enzyme in sites where it is highly active, such as skeletal muscle, thereby possibly decreasing the pools of BCKA and other BCAA-derived substrates that could be transported to the liver.

**Figure 5:**
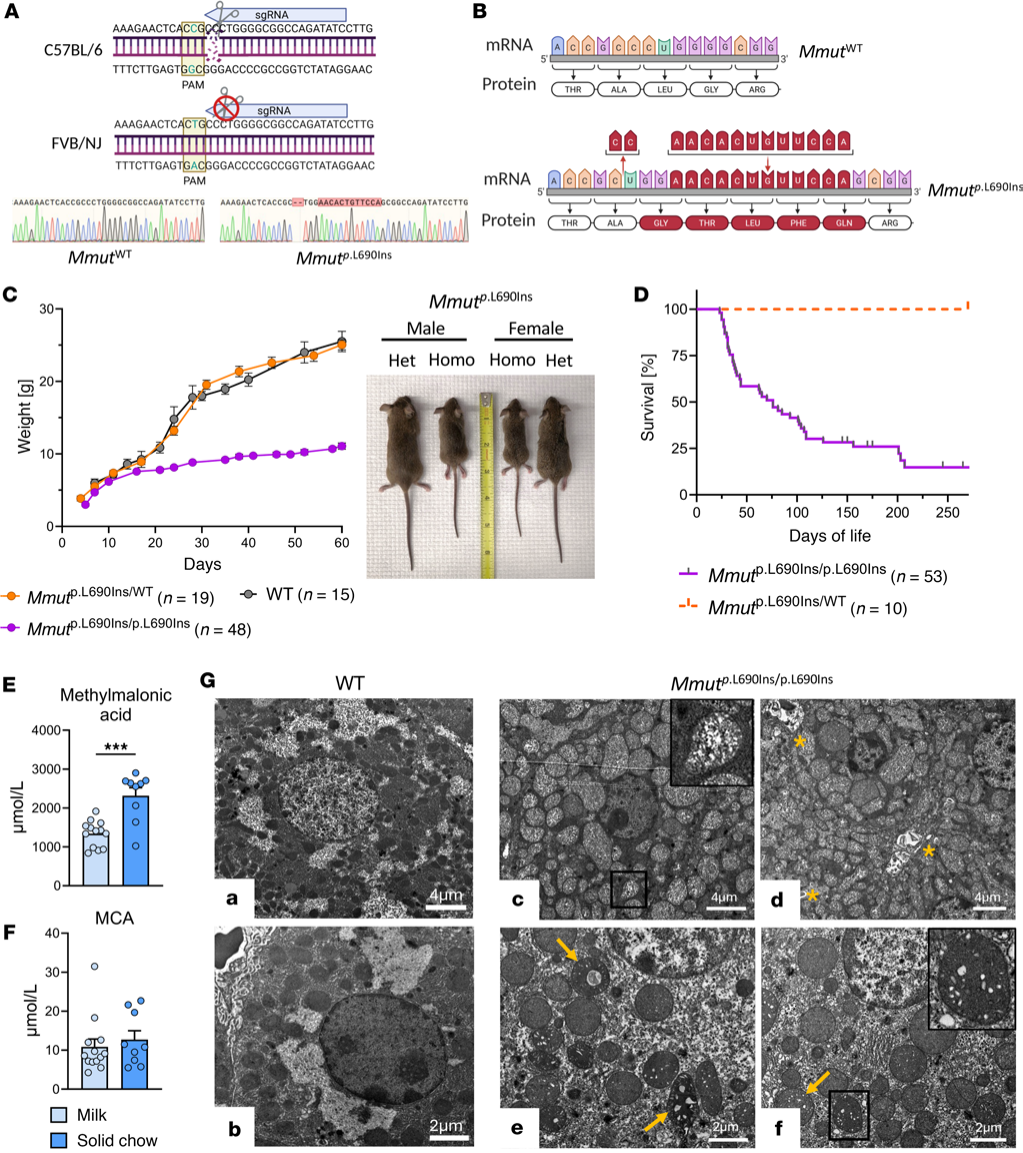
Characterization of an MMA mouse model, the *Mmut*^p.L690Ins/p.L690Ins^ mouse. (**A**) Schema of monoallelic CRISPR editing to generate the *Mmut*^p.L690Ins^ allele with representative Sanger sequencing results for wild-type and *Mmut*^p.L690Ins/p.L690Ins^ mice. (**B**) Schematic of RNA and amino acid sequence changes in the *Mmut*^p.L690Ins^ allele. (**C**) Body weights of homozygous, heterozygous, and wild-type mice and representative images for male and female *Mmut*^p.L690Ins/p.L690Ins^ (Homo) and *Mmut*^p.L690Ins/WT^ (Het) mice. (**D**) Survival of *Mmut*^p.L690Ins/p.L690Ins^ mice compared with heterozygous littermates. (**E** and **F**) Plasma methylmalonic acid (**E**) and MCA levels (**F**) in homozygous *Mmut*^p.L690Ins/p.L690Ins^ mice before and after transition to a solid chow diet following weaning. Results are mean ± SEM and analyzed by unpaired 2-tailed *t* test. “***” *P* ≤ 0.001. (**G**) Electron microscopy of mouse liver tissue. (a and b) Hepatocytes from *Mmut*^WT/WT^ mouse showing cytoplasmic glycogen deposits, well-spaced mitochondria, and visible organelles. (c) Hepatocyte from *Mmut*^p.L690Ins/p.L690Ins^ mouse with densely packed mitochondria. Cytoplasmic glycogen and other organelles are not apparent. Inset shows a single mitochondrion with pale or rarefied matrix. (d) Lamellated inclusion-like bodies (yellow asterisk) representing breakdown products within the cytoplasm. (e and f) Multiple abnormal mitochondria with ultrastructural changes, such as empty, microcystic, or macrocystic spaces within the matrix or central lamellated inclusions (yellow arrows, inset).

To study the effect of BCAT2 inhibition in a whole-body system, we generated a murine disease model of MMA by monoallelic CRISPR targeting to mitigate potential homozygous lethality (see Methods for more details). Founder animals were screened for novel in-frame mutations by genotyping and Sanger sequencing (Figure 5A). The *Mmut*^c.2067_2068del;^ ^2072G>A;2072_2073InsACACTGTTCCA^ mutant allele was identified as an in-frame insertion deletion that adds 3 amino acids and alters the existing amino acid sequence within the cobalamin binding domain (p.L690Ins, Figure 5B).

Homozygous *Mmut*^p.L690Ins/p.L690Ins^ mice were born normally and visibly indistinguishable from wild-type and heterozygote littermates until 2 weeks of age, when they began to show a distinct failure to thrive, with weights approximately 45% those of their wild-type and heterozygous littermates (Figure 5C). Homozygous pups perished as early as 25 days, with the largest proportion of lethality in the first 2 to 3 weeks following weaning. By day 75, fewer than 50% of homozygous mice survived (Figure 5D). *Mmut*^p.L690Ins/p.L690Ins^ mice displayed significantly elevated levels of methylmalonic acid (1,341 ± 332.4 μmol/L) in plasma that increased almost 2-fold to 2,315 ± 620.9 μmol/L within a week of transitioning to solid chow (Figure 5E). Another disease marker, MCA, was also elevated in the plasma (10.85 ± 7.163 μmol/L) at weaning but did not increase further upon transition to solid chow (Figure 5F). We also measured the concentration of FGF-21, which has shown promise as a new biomarker for MMA (39, 45). *Mmut*^p.L690Ins/p.L690Ins^ mice were found to have highly variable levels of FGF-21 compared with wild-type mice. FGF-21 accumulated over time in the mutant mice, rising to levels about 2.5 times higher at 6 months old compared with earlier time points of 1.5 and 3 months (Supplemental Figure 3A). Creatinine and blood urea nitrogen (BUN) levels were assessed as an indicator of renal function, with BUN slightly elevated above the normal range (Supplemental Figure 3B). Postmortem examination revealed a lack of visible abdominal fat depots and normal liver weight normalized to body weight compared with healthy, age-matched controls (Supplemental Figure 3C). Compared with wild-type mice, *Mmut*^p.L690Ins/p.L690Ins^ mice expressed less mutant MMUT protein in the liver. While MMUT was detected in heart, kidney, and muscle tissue of wild-type samples, the mutant protein was not visible on the Western blot (Supplemental Figure 3D). Histological evaluation of liver tissue from 4-week-old *Mmut*^p.L690Ins/p.L690Ins^ mice showed pale or vacuolated cytoplasm with fewer eosinophilic hepatocytes in portal and lobular areas compared with *Mmut*^WT/WT^ counterparts (Supplemental Figure 3E). Electron micrographs of liver from 4-week-old *Mmut*^p.L690Ins/p.L690Ins^ mice revealed densely packed mitochondria that obscured visibility of glycogen and prominent organelles, such as Golgi bodies and rough endoplasmic reticulum (Figure 5G). A spectrum of mitochondrial changes was observed in the *Mmut*^p.L690Ins/p.L690Ins^ hepatocytes, including microcystic and macrocystic spaces, pale or rarefied matrix, and concentric lamellar inclusion-like bodies, indicative of intramitochondrial degradation or mitophagy. These results are consistent with structural changes observed in livers of patients with MMA (39, 46). Electron micrographs from the kidney showed that *Mmut*^p.L690Ins/p.L690Ins^ renal tubular epithelial cells had densely packed mitochondria that obscured other cellular features. Electron-dense inclusions, disorganized cristae, and decreased density of the mitochondrial matrix were also observed (Supplemental Figure 3F). Evaluation of H&E-stained tissue via light microscopy revealed no abnormalities at this age (data not shown). These features recapitulate the disorganized cristae and enlarged mitochondria observed in patients with MMA (39, 46).

We rescued 1-week-old *Mmut*^p.L690Ins/p.L690Ins^ mice with a single intraperitoneal injection of 3.02 × 10^10^ vector genome copies per pup of an adeno-associated virus (AAV) vector expressing the wild-type human *MMUT* gene (AAV8.CB7.hMMUT) (37). Mice were completely rescued from lethality (Supplemental Figure 4A) and exhibited increased weights compared with untreated homozygous littermates (Supplemental Figure 4B). *Mmut*^p.L690Ins/p.L690Ins^ mice treated with AAV8.CB7.hMMUT also exhibited a significant reduction in plasma methylmalonic acid levels at approximately 1.5 months of age, 5 weeks following injection of the virus (Supplemental Figure 4C). In summary, these potentially novel *Mmut*^p.L690Ins/p.L690Ins^ mice recapitulate many of the biochemical and clinical presentations of MMA seen in human patients, survive into adulthood, and can be rescued with gene therapy.

### Evaluating the BCATi in a whole-body model of MMA.

**Figure 6:**
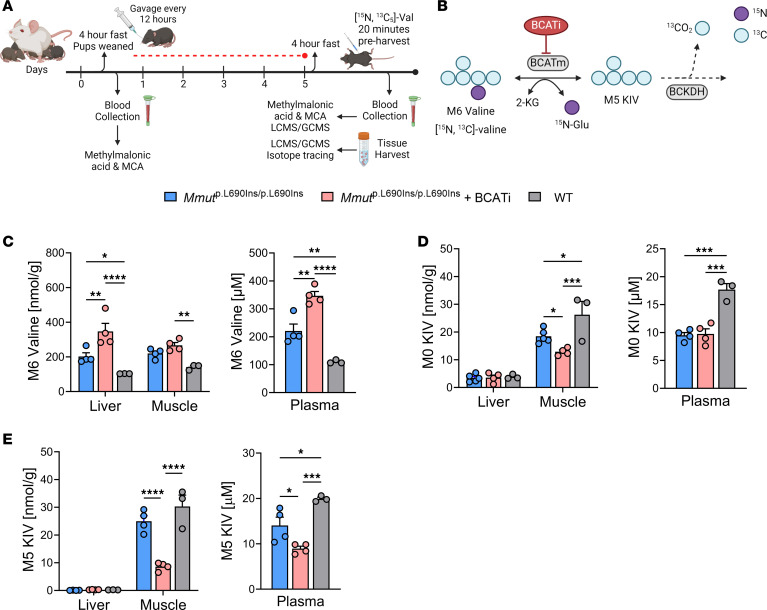
Evaluation of BCATi in an MMA mouse model. (**A**) Experimental design of in vivo metabolic flux study of BCATm inhibition. (**B**) Schematic of isotope tracing with labeled [^15^N, ^13^C_5_]valine. (**C**) Quantification of labeled M6 valine in liver, muscle, and plasma with M8 valine as internal standard. (**D** and **E**) Quantification of unlabeled M0 KIV and labeled M5 KIV in liver, skeletal muscle, and plasma with M8 KIV as internal standard. All results are mean ± SEM and analyzed by 1- or 2-way ANOVA, with statistical significance indicated by the symbols “*” *P* ≤ 0.05, “**” *P* ≤ 0.01, “***” *P* ≤ 0.001, “****” *P* ≤ 0.0001. LCMS, liquid chromatography–mass spectrometry.

*Mmut*^p.L690Ins/p.L690Ins^ mice exhibited clinical signs of MMA within the first few weeks of life, and severe disease seemed to be precipitated following a transition to solid chow after weaning that was accompanied by a marked increase in methylmalonic acid. We aimed to evaluate the effect of BCATi on metabolic flux through BCATm in a whole-body model as well as its effect on disease phenotypes. Thus, we chose to administer the drug at weaning in concert with the onset of disease. On the day of weaning, we collected blood and began administering BCATi (100 mg/kg, oral gavage) every 12 hours for 5 days to *Mmut*^p.L690Ins/p.L690Ins^ mice, with untreated *Mmut*^p.L690Ins/p.L690Ins^ and *Mmut*^WT/WT^ littermates as controls (Figure 6A). At the end of the 10-dose period, we injected the mice with [^15^N, ^13^C_5_]valine to quantify the effect of BCATi on metabolic flux of valine in liver, skeletal muscle, and plasma (Figure 6B).

We observed increased levels of labeled M6 valine in the liver and plasma of BCATi-treated *Mmut*^p.L690Ins/p.L690Ins^ mice compared with untreated *Mmut*^WT/WT^ and *Mmut*^p.L690Ins/p.L690Ins^ controls (Figure 6C). Consistent with a general effect on BCAA catabolism, *Mmut*^p.L690Ins/p.L690Ins^ mice treated with BCATi accumulated unlabeled valine, leucine, and isoleucine in muscle, liver, and plasma compared with untreated wild-type and *Mmut*^p.L690Ins/p.L690Ins^ mice (Supplemental Figure 5, A–C). Treatment with BCATi resulted in lower concentrations of both M0 (Figure 6D) and M5 (Figure 6E) KIV in skeletal muscle and plasma compared with untreated *Mmut*^WT/WT^ mice. Very little KIV (labeled or unlabeled) was present in liver tissue, consistent with the low level of BCATm activity in the murine liver, and BCATi treatment did not affect these levels (Figure 6, D and E). In addition to investigating this primary reaction, we quantified ^15^N labeling of leucine and isoleucine. This requires 2 distinct steps. First, ^15^N must be transferred from M6 valine to α-KG to form [^15^N]glutamate (M1 glutamate). M1 glutamate then serves as a nitrogen donor to transfer the ^15^N onto the branched-chain ketoacids in the reverse transaminase reaction to form M1 leucine, M1 isoleucine, or M1 valine (Supplemental Figure 6A). We observed lower levels of M1 glutamate labeling in skeletal muscle of BCATi-treated *Mmut*^p.L690Ins/p.L690Ins^ mice compared with untreated *Mmut*^p.L690Ins/p.L690Ins^ and wild-type mice (Supplemental Figure 6B), accompanied by reduced labeling of M1 leucine in skeletal muscle and isoleucine in both liver and skeletal muscle (Supplemental Figure 6C). In summary, these results establish that BCATi efficiently inhibits both the forward and reverse BCATm-mediated transamination reactions in *Mmut*^p.L690Ins/p.L690Ins^ mice.

**Figure 7:**
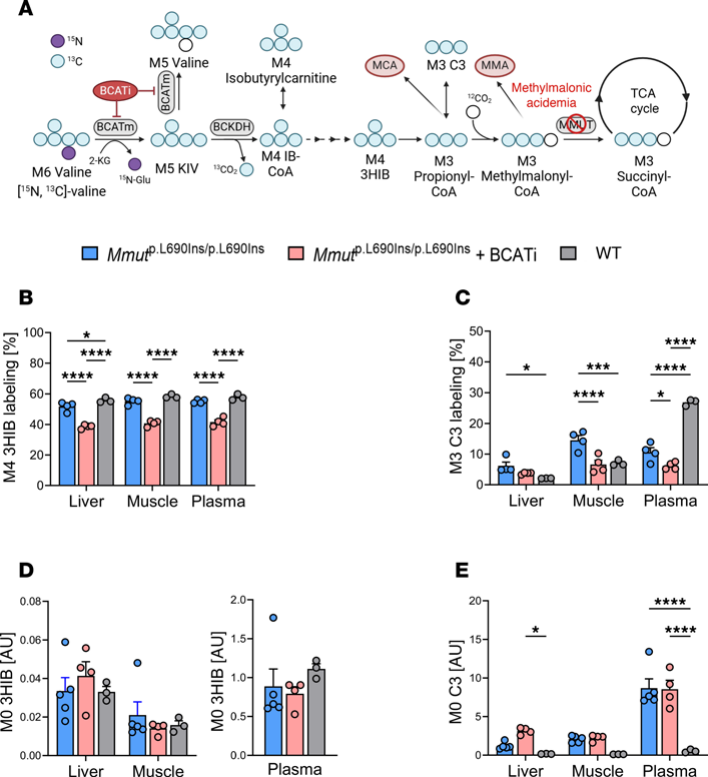
Measurement of distal metabolites in MMA mice following BCATi treatment. (**A**) Schematic of isotope tracing in *Mmut*^p.L690Ins/p.L690Ins^ and *Mmut*^WT/WT^ mice. (**B** and **C**) Percentage labeling of M4 3HIB (**B**) and M3 C3 acylcarnitine (**C**). (**D**) Relative quantification of M0 3HIB expressed as arbitrary units, representing peak areas for 3HIB relative to M8 2OHIV as an internal standard. (**E**) Relative quantification of M0 propionylcarnitine (C3) with D9 carnitine as an internal standard. All results are mean ± SEM and analyzed by 1- or 2-way ANOVA, with statistical significance indicated by the symbols “*” *P* ≤ 0.05, “***” *P* ≤ 0.001, “****” *P* ≤ 0.0001.

We next assessed the impact of BCATi on downstream catabolites M4 3HIB and M3 C3 in the murine disease model (Figure 7A). Following administration of M5 valine, significant decreases in percentage labeling of M4 3HIB were observed in BCATi-treated *Mmut*^p.L690Ins/p.L690Ins^ mice in liver, muscle, and plasma compared with untreated *Mmut*^WT/WT^ and *Mmut*^p.L690Ins/p.L690Ins^ controls (Figure 7B). Treatment with BCATi also reduced labeling of M3 C3 in plasma compared with untreated *Mmut*^p.L690Ins/p.L690Ins^ and *Mmut*^WT/WT^ mice and in muscle compared with the untreated *Mmut*^p.L690Ins/p.L690Ins^ group (Figure 7C).

In contrast, M0 3HIB pools were not significantly different between the 3 groups in any of the evaluated compartments (Figure 7D). In BCATi-treated *Mmut*^p.L690Ins/p.L690Ins^ mice, M0 C3 was elevated in liver and plasma compared with *Mmut*^WT/WT^ controls (Figure 7E), but C3 levels were not different in BCATi-treated compared with untreated *Mmut*^p.L690Ins/p.L690Ins^ mice. These data demonstrate that similar to our findings in isolated hepatocytes, inhibition of BCAT in the mouse model of MMA is not sufficient to decrease the level of a key distal metabolite (C3 acylcarnitine) that accumulates in liver in response to MMUT deficiency.

**Figure 8:**
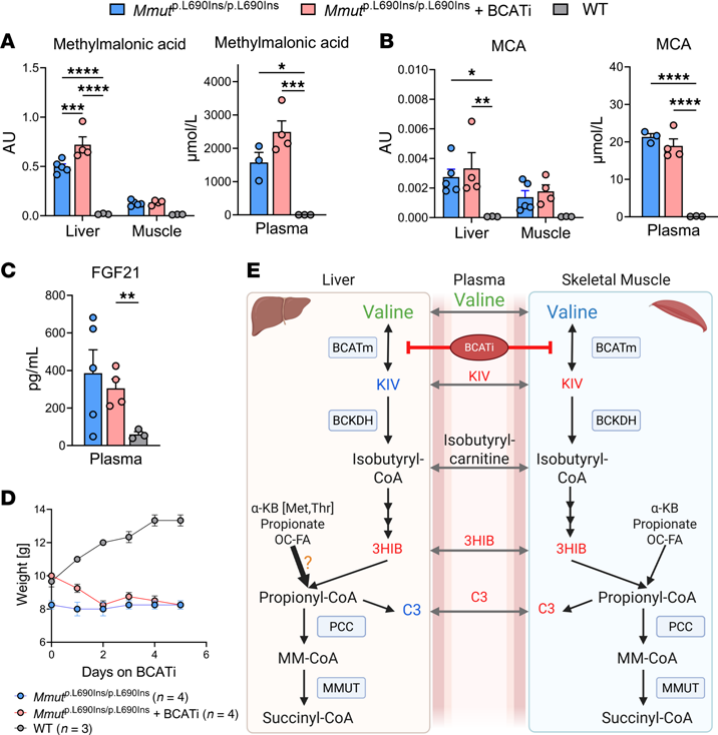
Disease markers in MMA mice following BCATi treatment. Quantification of (**A**) methylmalonic acid and (**B**) MCA at 4 weeks of age. (**C**) Plasma FGF-21 levels. (**D**) Body weights over course of 10-dose study. All results are mean ± SEM and analyzed by 1- or 2-way ANOVA. “*” *P* ≤ 0.05, “**” *P* ≤ 0.01, “***” *P* ≤ 0.001, “****” *P* ≤ 0.0001. (**E**) Graphical depiction of metabolic scheme in *Mmut*^p.L690Ins/p.L690Ins^ mice under the BCATi. The effects of the BCATi on measured metabolites in each compartment, compared with untreated *Mmut*^p.L690Ins/p.L690Ins^ mice, are indicated in blue (normal), green (increased), or red (decreased). Potentially increased contribution of propionyl-CoA from secondary sources as a result of BCATi is indicated by enlarged arrow and orange question mark.

As an independent measure of the effect of BCATm inhibition on the specific disease state of MMA, we quantified the levels of methylmalonic acid and MCA (Figure 8, A and B). Both treated and untreated *Mmut*^p.L690Ins/p.L690Ins^ mice exhibited significantly elevated levels of both methylmalonic acid and MCA in liver and plasma compartments compared with *Mmut*^WT/WT^ control mice. Treatment of *Mmut*^p.L690Ins/p.L690Ins^ mice with BCATi actually caused methylmalonic acid levels to increase further in liver relative to the untreated *Mmut*^p.L690Ins/p.L690Ins^ mice (Figure 8A). Additionally, treatment of *Mmut*^p.L690Ins/p.L690Ins^ mice with BCATi did not decrease plasma concentrations of an alternative MMA biomarker, FGF-21, compared with *Mmut*^WT/WT^ controls (Figure 8C). Finally, BCATi-treated *Mmut*^p.L690Ins/p.L690Ins^ mice also retained the decreased weight phenotype found in the untreated group (Figure 8D).

## Discussion

In this study we used human hepatocytes isolated from a patient with MMA during orthotopic liver transplantation as well as hepatocytes from a healthy human donor. Liver transplantation is not curative but markedly reduces the metabolic decompensation of patients with MMA and therefore is beneficial for severe forms of the disorder (47, 48). This demonstrates that MMA hepatocytes contribute to the pathogenesis of the disease and are a primary target of experimental therapeutics. Using isolated hepatocytes, our study establishes an authentic human model for MMA that recapitulates the known molecular features of the disorder, such as depletion of TCA cycle intermediates and accumulation of metabolites proximal to the impaired enzymatic activity. Moreover, tracing experiments demonstrate efficacy of our BCATm inhibitor in both the healthy and the disease-specific setting, with reduction of labeling of downstream catabolites from a stable isotope-labeled BCAA ([^15^N, ^13^C_5_]valine). However, in the isolated hepatocyte setting, BCATi treatment did not alter the concentration (pool size) of a key catabolic product of valine metabolism, C3 acylcarnitine, suggesting that the main proportion of these metabolites are derived from sources other than valine flux through BCATm. Consistent with this idea, BCATm activity was very low in both healthy and MMUT-deficient human hepatocytes, such that relatively high concentrations of [^15^N,^13^C_5_]valine were required to observe flux to distal products. Even at these high substrate doses, labeling of TCA cycle intermediates was essentially undetectable, whereas provision of [^13^C_5_]KIV allowed clear labeling of these metabolites. The low BCATm activity in the cultured human hepatocytes reflects the low activity in the human liver. Thus, while these studies provide valuable information about the BCATi and metabolic differences between healthy and MMUT-deficient hepatocytes, they do not allow us to test the idea that BCAT inhibition in other organs, namely the skeletal muscle, could lower toxic catabolites in the liver and be useful for the therapy of MMA. Also, hepatocytes begin to dedifferentiate and change their gene expression profile in culture, such that mitochondrial structure and function specific to MMA might not be recapitulated in such in vitro studies, limiting their utility.

To address these limitations and further evaluate the therapeutic potential of BCATi in an in vivo setting, we developed a mouse model of MMA, *Mmut*^p.L690Ins/p.L690Ins^, that closely mimics the human disease. We hoped that this model would allow us to evaluate the impact of inhibition of BCATm across all the tissues in which it is expressed, including high levels in skeletal muscle and kidney. We reasoned that inhibition of BCATm in these extrahepatic tissues could reduce levels of circulating branched-chain ketoacids or other substrates contributing to accumulation of toxic metabolites upstream of the MMUT lesion in liver. When we applied our BCATi to this MMA disease model, we were again able to verify efficient BCATm inhibition but with no reduction in the concentration of distal BCAA catabolites, such as M0 3HIB or M0 C3. Moreover, BCATi treatment did not lower the levels of disease markers, such as methylmalonic acid or FGF-21. Finally, key disease phenotypes of the *Mmut*^p.L690Ins/p.L690Ins^ mice, such as weight loss, were not rescued by BCATi, in contrast with complete rescue in response to replacement of the defective *Mmut* gene with the normal human *MMUT* gene via an AAV construct, as shown here and previously in other murine MMA models (37).

Our tracing experiments in vivo unveil complex organ-specific BCAA catabolism. Labeled and unlabeled KIV were present at very low levels in the liver relative to plasma or muscle, and those levels were unaffected by genotype or presence or absence of BCATi treatment, consistent with the very low levels of BCATm in murine liver. This occurred despite accumulation of M0 and M6 valine in liver in response to BCATi treatment of *Mmut*^p.L690Ins/p.L690Ins^ mice, demonstrating effective delivery of the labeled substrate to the target tissue. In contrast, BCATi treatment of *Mmut*^p.L690Ins/p.L690Ins^ mice decreased both the pool size (M0) and labeling (M5) of the KIV pool in muscle relative to either vehicle-treated *Mmut*^p.L690Ins/p.L690Ins^ or wild-type mice. These results demonstrate a clear effect of BCATi to inhibit BCATm in the muscle, where it is known to be highly expressed, with little effect in liver, which has very low levels of expression of the enzyme. Interestingly, the percentage of 3HIB labeling in the liver was very high (~50%) and comparable to the plasma and muscle. This is consistent with a shunt of 3HIB or another intermediate from the muscle to the liver, rather than as a function of hepatic valine catabolism. Consistent with this idea, BCATi reduced the percentage labeling of the 3HIB pool in liver to almost exactly mirror changes in labeling of the muscle and plasma 3HIB pools. Nevertheless, total pools (labeled and unlabeled) of 3HIB and its downstream catabolite C3 acylcarnitine were not reduced by BCATi treatment of *Mmut*^p.L690Ins/p.L690Ins^ mice in any compartment analyzed. Although labeled C3 in the muscle and plasma was reduced, the labeling percentage was low (<20%), implying the generation of C3 by alternative sources, such as propionate, threonine, methionine, or OC-FA (Figure 8E).

Labeling of 3HIB of approximately 50%, accompanied by much lower percentage labeling of the C3 pool, is consistent with important contributions to the C3 pool from unlabeled, non-BCAA-derived substrate sources. In alignment with this interpretation, treatment of *Mmut*^p.L690Ins/p.L690Ins^ with BCATi did not lower the key metabolic biomarkers of MMA, methylmalonic acid or MCA. Also consistent with these findings, the labeling of methylmalonic acid and MCA after injection of [^15^N, ^13^C_5_]valine was too low for quantification, suggesting very minimal utilization of valine as a source of methylmalonic acid and MCA production. However, we must consider that our flux analyses are only a snapshot after 20 minutes and do not reflect steady-state kinetics. Also, we did not analyze metabolism of other propionate precursors, such as OC-FA, cholesterol catabolites, gut propionate, or the amino acids isoleucine, methionine, and threonine, which could be the subject of future studies. The total amino acid contribution for propionyl-CoA production has been estimated to be 50% in patients with MMA (*n* = 3) and propionic acidemia (*n* = 3) with a range from 25% to 66% (49) and 5%–40% in another set of 5 patients with MMA (50). Both studies had substantial interindividual variability and, as noted by the authors, are an overestimation, since not all threonine and methionine degradation yields propionyl-CoA. Our data indicate that valine is not a crucial contributor to propionyl-CoA formation. Moreover, while the BCATm inhibitor had effective target engagement in the in vivo setting, this tool had no impact on the key biochemical markers of MMUT deficiency, methylmalonic acid and MCA.

A clinical report from the “domino transplantation” literature supports our findings (51). This technique uses the explanted and diseased liver for transplantation into another secondary patient to address the organ shortage and urgent need in some patients. In this study, a liver from a patient with a severe form of BCKDH (Figure 1) deficiency, called maple syrup urine disease, was transplanted into a patient with propionic acidemia (51) with a deficiency at the propionyl-CoA carboxylase step in propionyl-CoA catabolism (Figure 1). Hence, a liver with a proximal block in BCAA catabolism (BCKDH) was transplanted into a patient with a distal BCAA disorder (PCC block). Interestingly, the transplanted liver was not able to normalize the C3 levels of the patient; C3 levels were about 100 times higher than the upper limit of the reference range. This clinical case report is a 1-patient observation and differs in many ways from our study. The blocks in BCAA catabolism were at different steps, and our studies are in an MMA mouse model. Nevertheless, this clinical observation is in agreement with our insight that valine catabolism is not a major supplier of distal BCAA catabolites. Hence, inhibition of a proximal step in BCAA catabolism, such as BCATm, is unlikely to be a successful strategy for distal BCAA disorders. This contrasts with other amino acid degradation disorders, where proximal inhibition of catabolic pathways is either the standard of care (tyrosinemia type I) (19) or has recently emerged as a valuable therapeutic approach (glutaric aciduria type I) (21).

Our study highlights the importance of deploying multiple and complementary models for testing new drug targets for treatment of metabolic disorders. While the BCATi used here efficiently inhibited the human (and murine) target enzyme, it failed to reduce the clinical symptoms in a potentially novel MMA mouse model. Also, the metabolic crosstalk between organs cannot be mimicked in a dish, and the value of studies in experimental animal models or humans is made clear by our findings. Probably the most important lesson is that a detailed understanding of the underlying metabolic pathology is a necessity for the development of new therapeutic strategies.

## Methods

### Sex as a biological variable.

Our study examined male and female animals for the characterization of the potentially novel *Mmut*^p.L690Ins/p.L690Ins^ mouse strain, and similar findings are reported for both sexes. To keep experiments standardized (weight differences between sexes), we used only male mice for isotope tracing.

### Inhibition of recombinant hBCATc and hBCATm.

Inhibition of recombinant human BCATc (hBCATc) and hBCATm was assessed using a cell-free enzyme-coupled fluorescence assay. BCATi was prepared as up to a 10 mM stock solution using DMSO as the vehicle. We generated 10-point dose-response curves using the Echo-550 (Labcyte) acoustic dispenser. BCATi source places were made by serially diluting stocks to generate a final 384-well assay test concentration range from 3 μM to 0.0001 μM. A total of 10 μL of 4× hBCAT1 and hBCAT2 enzyme stocks were added to the prepared assay plate and incubated at room temperature, protected from light, for either 10 or 60 minutes. After the incubation period, 20 μL of assay buffer plus 2× substrate was added to all wells (final substrate concentration was 300 μM l-leucine and 250 μM α-KG). The enzyme reaction was then incubated for 10 minutes at room temperature before halting with the addition of 5 μL of 0.6N HCl and incubated for 1 minute. Next, 5 μL of 1 M Tris (pH 8.0) was added to neutralize pH. Then, 12.5 μL of the quenched assay volume was transferred to a white, 384-well plate, and 12.5 μL of glutamate detection reagent was added (prepared per Promega Glutamate-Glo kit instructions). Plates were then incubated for 30 minutes at room temperature, protected from light. Following the incubation period, luminescence was read on BMG Novostar and relative light units were measured. IC_50_ values were calculated in ActivityBase, using a 4-parameter fit equation.

### Liver tissue procurement and hepatocyte isolation.

Hepatocytes were isolated by 2-step collagenase perfusion method as previously described (52). In brief, we cannulated the largest portal veins with a silicon tubing system connected to a peristaltic pump, then flushed the liver with ice-cold basic perfusion solution (BPS: 10 mM HEPES buffer), followed by perfusion with BPS containing 0.5 mM EGTA to prevent the formation of blood clots. The liver was then perfused with warm collagenase solutions (2 mg/dL collagenase) until the organ became soft. We cut the liver into small pieces (2–3 cm^3^) and released hepatocytes into the solution by applying minor shear stress (with forceps) on the pieces. Hepatocytes were immediately washed in ice-cold BPS containing 0.5% BSA and centrifuged (3 times at 50*g*, 5 minutes). Viability was assessed by trypan blue exclusion. Hepatocytes were stored in NG5A cryopreservation solution (ChemQ).

### Isotope tracing in primary culture of hepatocytes.

Frozen human hepatocytes were thawed and spun down at 90*g* for 3 minutes before being resuspended in 10 mL of plating media (VitroPrep CQ-HPM-250). One million cells/well were seeded on Matrigel-coated 12-well plates and incubated at 37°C for 3 hours. After 3 hours, plating media were aspirated and the wells washed twice with 1 mL sterile 1× PBS before adding 2 mL of secretion buffer (10× salt stock, 1 M HEPES, 0.25 M CaCl_2_, 0.2% BSA, and 25.5 mM NaHCO_3_), 800 μM [^15^N, ^13^C_5_]valine, and 3 nM, 100 nM, or 3,000 nM BCATi *or* 800 μM [^13^C_5_]KIV and 100 nM, or 3,000 nM BCATi to each well. Each condition was assayed in triplicate. Cells were incubated for 1 hour at 37°C before removing media, immersing the plate in liquid nitrogen, and stopping the reaction with 1.5 mL methanol and 20 μL 0.05 μM norvaline as an internal standard for quantification of intermediates.

### Gas chromatography–MS for metabolite profile.

We profiled the metabolic changes in cells, organs, and plasma using our previously published gas chromatography–MS method (53, 54). Briefly, for the in vitro study, 1 million cells were spiked with 0.2 nmol of norvaline and 0.2 nmol M9 carnitine and then subjected to extraction through the standard Folch method with 400 μL methanol, 400 μL H_2_O, and 400 μL chloroform. For the in vivo study, 20 mg of tissue or 20 μL plasma was spiked with 2 nmol of [^2^H_4_,1,2,3,4-^13^C_4_]KIV (M8 KIV), [^2^H_4_,1,2,3,4-^13^C_4_]2-hydroxyisovalerate (M8 2OHIV), D8 valine (M8 valine), [^13^C_6_]isoleucine (M6 isoleucine), and 0.2 nmol M9 carnitine as internal standards. Then 500 μL methanol was added and vortexed, followed by 500 μL acetonitrile being added and vortexed. Samples were centrifuged for 20 minutes at 3,000*g*. The upper phase, approximately 300 μL in volume, was transferred to a fresh Eppendorf vial and subsequently evaporated using nitrogen gas. The resulting dried residues underwent sequential derivatization with methoxylamine hydrochloride and *N*-*tert*-butyldimethylsilyl-*N*-methyltrifluoroacetamide (TBDMS). Specifically, 40 μL of methoxylamine hydrochloride (2% w/v in pyridine) was added to the dried residues, followed by incubation for 90 minutes at 40°C. Subsequently, 20 μL of TBDMS with 1% *tert*-butylchlorodimethylsilane was added, and the mixture was incubated for an additional 30 minutes at 80°C. The derivatized samples were then centrifuged for 10 minutes at 12,000*g*, and the supernatants were transferred to gas chromatography (GC) vials for further analysis. For GC/MS analysis, we employed an Agilent 7890B GC system with an Agilent 5977A Mass Spectrometer, following the methodology described in our previous work (53, 54). Specifically, 1 μL of the derivatized sample was injected into the GC column. The GC temperature gradient began at 80°C for 2 minutes, increased at a rate of 7°C per minute to 280°C, and was maintained at 280°C until the 40-minute run time was completed. The ionization was conducted via electron impact at 70 eV, with helium flow at 1 mL/min. Temperatures of the source, the MS quadrupole, the interface, and the inlet were maintained at 230°C, 150°C, 280°C, and 250°C, respectively. Mass spectra (*m/z*) in the range of 50 to 700 were recorded in mass scan mode.

### LC-MS/MS for acylcarnitine profile.

Tissue or plasma acylcarnitines were methylated and profiled using a modified liquid chromatography–tandem MS (LC-MS/MS) method (54). The tissue or plasma sample extracts (300 μL) from the previous sample preparation were completely dried using nitrogen gas. The dried residues were then methylated with a 3 M HCl methanol solution (100 μL) at 50°C for 25 minutes. After methylation, the samples were once again dried completely using nitrogen gas and then reconstituted in 20 μL of methanol and 60 μL of water. The derivatized samples were subsequently analyzed using an LC-QTRAP 6500^+^-MS/MS (Sciex). A gradient HPLC method with 2 mobile phases (mobile phase A was 98% water with 2% acetonitrile and 0.1% formic acid, and mobile phase B was 98% acetonitrile with 2% H_2_O and 0.1% formic acid) was adopted to run with an Agilent Pursuit XRs 5 C18 column (150 × 2.0 mm). The gradient started with 0% B within the first 2 minutes and then increased to 80% at 13 minutes. The column was washed out with 90% B for 4 minutes and equilibrated with initial condition (2% B) for 5 minutes before the next injection. The flow rate was 0.4 mL/min, and the column oven was set at room temperature. The injection volume was 2 μL. The parameters for Sciex QTRAP 6500^+^ MS were optimized as follows: DP: 33 V, EP: 10 V, CXP: 10 V, source temperature: 680°C, gas 1: 65 PSI, gas 2: 65 PSI, curtain gas: 35 PSI, CAD: 10, and ion spray voltage: 5,500 V. The Q1 of all the methylated acylcarnitines was scanned from *m/z* 218 to *m/z* 444 with the same fragment (Q3) at *m/z* 99. l-Carnitine had the ion transition of Q1 (*m/z* 176) and Q3 (*m/z* 85 or *m/z* 117). M9 carnitine had the shifted Q1 at *m/z* 179 or *m/z* 185 with the same Q3 at *m/z* 85 or *m/z* 117.

### Generation of the Mmut^L690Ins^ mouse strain.

We designed a single-guide RNA (sgRNA) within exon 12 of *Mmut* that has an “in PAM” polymorphism in the genome of FVB/NJ mice (Figure 5A). Pronuclear-stage mouse embryos of a mixed C57BL/6 FVB/NJ background were electroporated with the SpCas9/sgRNA ribonucleoprotein (RNP) (55). Benchling was used to identify *S*. *pyogenes* gRNA sequences in the cobalamin binding domain of C57BL/6 *Mmut*. Candidate sgRNAs were cross-referenced against the *Mmut* sequence from FVB/NJ mice to screen for “in PAM” polymorphisms. We selected an sgRNA (5′-GATATCTGGCCGCCCCAGGG-3′) targeting exon 12 that harbors a naturally occurring “in PAM” polymorphism in the FVB/NJ *Mmut* sequence. Additional sgRNAs flanking the region were designed for validation purposes. In vivo validation was performed via SLiK method as previously described (56, 57). In short, sgRNAs were annealed and ligated into pShuttle-SLiK with *Xba*I restriction enzyme and injected in pairs into C57BL/6 mice via hydrodynamic tail vein injection. Liver tissue was harvested 72 hours later, and genomic DNA was extracted. On-target cutting was confirmed by PCR followed by Sanger sequencing with the following PCR primers: forward 5′-ACTAGTGAGATGCACATAAGTGGG-3′ and reverse 5′-CATATGTATCATGGTGGGTGGGG-3′.

Generation of mice carrying the c.2067_2068del; 2072G>A;2072_2073InsACACTGTTCCA mutation was performed by the Duke Transgenic Mouse Facility using CRISPR-EZ technology (55). ~4-week-old female FVB/NJ mice were superovulated before immediate mating with fertile C57BL/6 male mice. Pronuclear-stage embryos were collected (at 0.5 dpc) and exposed to acidic Tyrode’s solution for ~30–40 seconds to partially erode (30%) the zona pellucida to enhance RNP delivery. Cas9/sgRNA RNPs, assembled in vitro, were combined with the embryos in an electroporation cuvette and subjected to a series of six 3 ms 30 V electrical pulses. Electroporated embryos were transferred to the oviduct of 0.5 dpc pseudopregnant CD1 female mice. Heterozygous founder mouse genotyping was performed on genomic DNA extracted from tail tissue via Sanger sequencing using primers 5′-ATTGATGTTCATTGTGTCAGTAGC-3′ (forward) and 5′-TGACCTACTGAGCCGCCTAAGAAC-3′ (reverse). Founder mice were crossed out to C57BL/6 mice for 3 generations to confirm transmittance of the mutant allele. Further offspring genotyping was performed by Transnetyx. Heterozygous *Mmut*^p.L690Ins/WT^ mice appear normal compared with wild-type (*Mmut*^WT/WT^) littermates, and homozygous *Mmut*^p.L690Ins/p.L690Ins^ mice are characterized in detail in the Results section.

### In vivo experimental methods.

Mice were maintained under a standard 12-hour dark/12-hour light cycle with water and regular chow provided ad libitum. Temperature and humidity were controlled.

~3-week-old *Mmut*^p.L690Ins/p.L690Ins^, *Mmut*^p.L690Ins/WT^, and *Mmut*^WT/WT^ mice were removed from breeder cages and fasted for 4 hours before collecting blood in green-top collection tubes via retroorbital bleeding. Mice received 200 μL subcutaneous saline and were left to recover on a heating pad for 30 minutes with regular chow and moistened fines added to the cage. At ~4 weeks, mice were again fasted 4 hours and had blood collected. Whole blood was centrifuged at 3,000*g* for 10 minutes to isolate plasma. Mice were euthanized following bleeding. Tissue from liver, kidneys, spleen, heart, lung, brain, and skeletal muscle was collected and both snap-frozen in liquid nitrogen and fixed overnight in 10% formalin.

~1-week-old male *Mmut*^p.L690Ins/p.L690Ins^ mice were administered 3.02 × 10^10^ vector genome copies of AAV8.CB7.hMMUT viral rescue vector, as shown before (37), via intraperitoneal injection. Mice were weighed regularly. At 5 weeks following injection of the virus, ~1.5-month-old mice were fasted 4 hours before collecting blood in green-top collection tubes via retroorbital bleeding. Mice received 200 μL subcutaneous saline and were left to recover on a heating pad for 30 minutes.

### Isotope tracing with BCATm inhibitor in Mmut^p.L690Ins/p.L690Ins^ mice.

~3-week-old male *Mmut*^p.L690Ins/p.L690Ins^ pups were removed from the breeder cage in the morning and fasted 4 hours. Following the 4-hour fast, blood was collected in green-top tubes via retroorbital bleeding. Mice were administered 200 μL saline subcutaneously and left to recover on a heating pad for 30 minutes with regular chow and moistened fines added to the cage. Starting at 6:30 pm and following at regular 12-hour intervals, mice were gavaged with 10 μL/g 0.5% methylcellulose/0.5% Tween 80 solution or 100 mg/kg BCATi (10 mg/mL solution of BCATi in 0.5% methylcellulose/0.5% Tween 80). After a total of 10 doses (4.5 days), mice were fasted for 4 hours immediately following the 10th dose. At 3 hours and 40 minutes into the fast, mice were given an intraperitoneal injection of 100 mg/kg [^15^N, ^13^C_5_]valine in saline. At 15 minutes following the isotope injection, mice were put under isoflurane anesthesia, and blood was collected in green-top tubes via retroorbital bleeding at 20 minutes after isotope injection. Whole blood was centrifuged at 3,000*g* for 10 minutes to isolate plasma. Mice were euthanized and liver and skeletal muscle from the quadriceps were immediately snap-frozen at 25 minutes after isotope injection and stored at –80°C. Liver and kidney tissue samples were fixed overnight in 4% paraformaldehyde, 10% formalin, and Trump’s fixative for future histological analyses. Kidney, spleen, heart, lung, and brain tissue were also snap-frozen and preserved in 4% paraformaldehyde.

### Blood analyses.

Plasma methylmalonic acid and MCA were measured by UPLC-MS/MS according to standard operating procedures (performed by Duke University Health System’s CLIA/CAP-certified Biochemical Genetics Laboratory). In brief, a fixed volume of plasma or serum (50 μL) was spiked with internal standard. Disulfide bonds were then reduced with tris(2-carboxyethyl)phosphine hydrochloride and proteins precipitated with 10% sulfosalicylic acid. The supernatant was evaporated to dryness under a gentle stream of nitrogen at 35°C. Upon dryness 200 μL of 3N butanolic HCl was added, and samples were derivatized at 65°C for 90 minutes to promote butyl esterification, evaporated to dryness under nitrogen at 35°C, and reconstituted in a solvent matrix of acetonitrile/deionized H_2_O (50/50 v/v) with 0.1% formic acid. Reconstitutes were analyzed by UPLC-MS/MS in positive-ionization mode using a Xevo TQD tandem quadrupole mass spectrometer equipped with an Acquity UPLC system (Waters Corp.). Separation of all analytes was achieved using an Acquity UPLC BEH C18, 2.1 mm × 100 mm, 1.7 μm column, and a linear gradient. The target analytes were detected by selected reaction monitoring. The peak area ratio of analyte to its internal standard was converted to a concentration using a 1/*x* weighted linear fit calibration curve (58).

Creatinine and BUN measurement was performed by the Research Services Laboratory in the Center for Comparative Medicine at Baylor College of Medicine using plasma from heparinized collection tubes.

Plasma FGF-21 levels were quantified via ELISA (BioVendor R&D, RD291108200R). Plasma was diluted 3 times in dilution buffer before loading 100 μL in microplate wells precoated with polyclonal anti-mouse FGF-21 antibody and incubated for 1 hour, shaking at 300 rpm. Wells were washed 3 times with wash solution (0.35 mL per well) before adding 100 μL of biotin-labeled polyclonal anti-mouse FGF-21 antibody and incubated on a shaker for 1 hour with captured FGF-21. Wells were washed with wash buffer 3 times before adding 100 μL streptavidin-HRP conjugate to each well and incubating for 30 minutes on the microplate shaker. Primary and secondary antibodies are provided as part of the ELISA kit. The wells were washed 3 times with wash solution before adding 100 μL of substrate solution to each well and covering the plate. The plate was incubated for 10 minutes at room temperature before adding 100 μL of stop solution. Within 5 minutes of stopping the reaction, absorbance of each well was determined using a microplate reader set to 450 nm, with reference wavelength set to 630 nm.

### Histology.

Fresh liver and kidney tissue samples were fixed in 10% formalin and processed on the Tissue-Tek VIP (Sakura). They were then embedded, cut, and stained on a Leica Autostainer XL with a standard hematoxylin and eosin protocol. In short, slides were deparaffinized and rehydrated. Slides were stained with hematoxylin solution for 3 minutes, washed with water, and then stained with eosin Y solution for 2 minutes. Slides were then dehydrated and mounted.

### Electron microscopy.

Liver and kidney samples were fixed and stored in Trump’s glutaraldehyde. Samples were washed in 0.1 M Trump’s buffer for 20 minutes, then in 1% osmium tetroxide in 0.1 M Trump’s Buffer for 1 hour before 3 consecutive 10-minute washes in water. Tissue was dehydrated in increasing concentrations of ethanol from 50% to 100%. Following dehydration, samples were cleared for 20 minutes in propylene oxide. This step was repeated before infiltration. Samples were first incubated for 50 minutes in a 50% stock resin/50% propylene oxide mixture. Stock resin is composed of 50% Embed 812 and 50% dodecenyl succinic anhydride. Samples were then placed for 70 minutes in 70% stock resin/25% propylene oxide before a final 30-minute incubation in 100% stock resin. The tissue was embedded in stock resin mixed with the catalyst 2,4,6-tri(diethylaminoethyl phenol) and left overnight at 100°C to harden. Cutting was performed using a Leica EM UC7 and DiATOME ultra 45° diamond knife. Completed tissue blocks were trimmed using a single-edge razor blade and 0.5 μm sections cut. The 0.5 μm sections were dried on a microscope and stained with 2 solutions: Solution A — 1% Methylene Blue, 1% Azure II, and 1% sodium borate — and Solution B — 0.5% Basic Fuchsin. Ultrathin sections (80–90 nm thick) were picked up on 150 copper mesh grids. The grids were stained with 2 solutions: Solution A — UranyLess EM Stain — and Solution B — Lead Citrate. All images were taken on a JEOL 1400 Flash TEM fitted with an AMT digital imaging system. Captured images were reviewed and interpreted by a pediatric pathologist.

### Western blot.

A total of 20 mg of fresh-frozen liver, kidney, heart, lung, brain, and skeletal muscle tissue was homogenized with 1 mL ice-cold RIPA buffer (Sigma-Aldrich, R0278) with 1× Proteinase inhibitor (Halt Protease and Phosphatase Inhibitor Cocktail, 100×, Thermo Fisher Scientific, 78440). A total of 5 μg of homogenized samples were premixed with loading buffer containing NuPAGE LDS Sample Buffer and NuPAGE Reducing Agent and heated for 10 minutes at 100°C. The premixed samples were loaded into wells of a NuPAGE 4% to 12% Bis-Tris Gel (Invitrogen) with MOPS SDS Running Buffer. Following transfer to a PVDF membrane, the membrane was blocked (EveryBlot Blocking Buffer, BIO-RAD) for 30 minutes and incubated overnight at 4°C with primary antibodies. Rabbit anti-MMUT (Abcam, ab134956) and mouse anti–β-actin (Sigma-Aldrich, A1978) were diluted 1:1,000 in blocking buffer. After washing, membranes were incubated for 1 hour at room temperature with donkey anti-rabbit HRP secondary antibody (Jackson ImmunoResearch, 711-035-152; diluted 1:5,000). Chemiluminescence detection was performed by incubating the membrane with Super Signal West Femto solution (Thermo Fisher Scientific, 34096) and imaging with ChemiDoc MP Imaging System (BIO-RAD, 12003154).

### Statistics.

All numerical data were evaluated using Prism software version 10.2.2 (GraphPad). One-way ANOVA with Tukey’s multiple comparisons test was used to determine the differences between in vitro conditions within either the control or the MMA hepatocyte groups. Two-way ANOVA with Tukey’s multiple comparisons test was used to determine the difference between the control and MMA groups within each culture condition. Statistical analysis of quantitative data collected from in vivo experiments was performed using 1- or 2-way ANOVA with Tukey’s multiple comparisons test. In quantitative analyses where only 2 groups were compared, significance was evaluated with unpaired 2-tailed *t* test. *P* ≤ 0.05 was considered a statistically significant difference.

### Study approval.

For human hepatocyte isolation, written informed consent was received prior to participation. The study was approved by the Institutional Review Board at Duke University. All animal work was performed under approval of the Duke University Institutional Animal Care and Use Committee.

### Data availability.

All data generated or analyzed during this study are included in the manuscript. No additional datasets were used or generated. Values for all data points found in graphs are in the Supporting Data Values file.

## Author contributions

CBN, KDB, GFZ, DK, and MGH designed the experiments. KDB, CBN, and MGH wrote the paper and integrated input from the other authors. MC provided conceptual input. MGH generated the MMA mouse model. MB and BBC helped with knockout design and crossing of the mice. MGH, TC, XL, and YM performed in vivo experiments. KDB isolated the human hepatocytes. YM performed in vitro experiments. MGH, SC, TC, XL, and YM helped with harvesting of mice. GFZ and HM performed metabolomics and stable isotope-based flux analyses. SC and TC did antibody-based assays. DHLT and KRP did the histopathological analysis. SPY, JB, and ARS did the MMA and MCA analysis. RJC and CPV provided the AAV vector and helped with conceptual input for experiments. MGH generated the new animal model and so is named first among the two co–first authors and performed the animal and in vitro cellular studies in collaboration with GFZ, who designed and executed the metabolic flux experiments, including MS-based analyses.

## Supplementary Material

Supplemental data

Unedited blot and gel images

Supporting data values

## Version 1 09/09/2025

Electronic publication
