# Supplementary material for: Dissecting the effect of mitochondrial BCAT inhibition in methylmalonic acidemia
Source: JCI Insight. 2025 Sep 9;10(17):e187758. doi: 10.1172/jci.insight.187758 (PMC12487681; doi:10.1172/jci.insight.187758)
Supplement: Supplemental data [file jciinsight-10-187758-s047.pdf]

A.

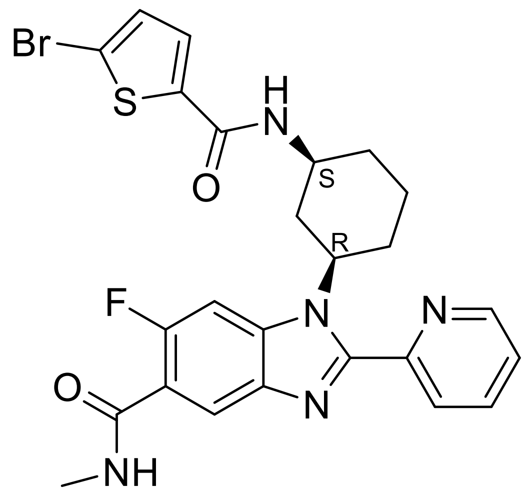

1-((1R,3S)-3-(5-bromothiophene-2-carboxamido)cyclohexyl)-6-fluoro-N-methyl-2-(pyridin-2-yl)-1H-benzo[d]imidazole-5-carboxamide

B.

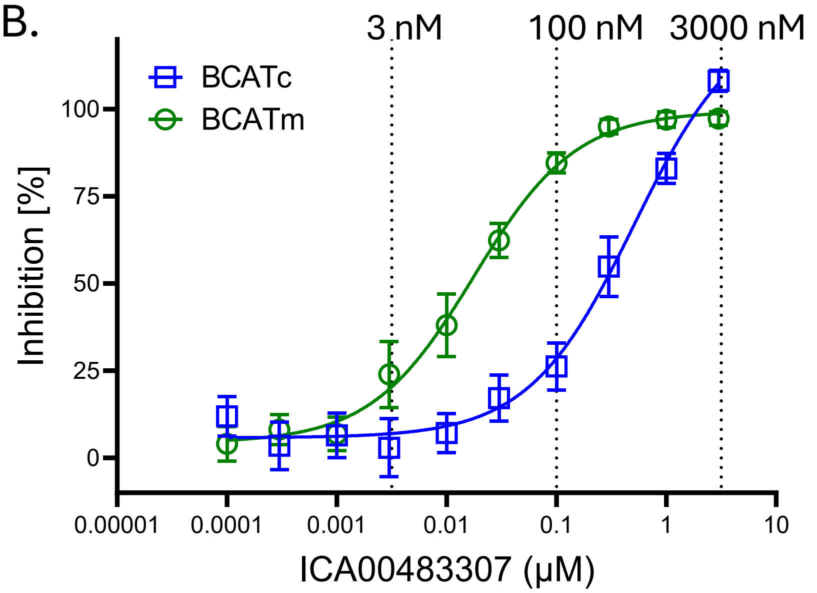

	BCATc	BCATm
HillSlope	0.91	0.93
IC50 (nM)	508.6	17.6
95% CI of IC50	340 to 1038	14.1 - 21.8

Supplemental Figure 1. Characterization of a BCATm Inhibitor (BCATi) A. Molecular structure and IUPAC name of BCATi (ICA00483307) **B.** Inhibition of recombinant hBCATc and hBCATm was determined by luminescent detection of glutamate production (see methods). Concentrations evaluated by in vitro assays 3, 100 and 3000 nM are marked by vertical dashed lines.

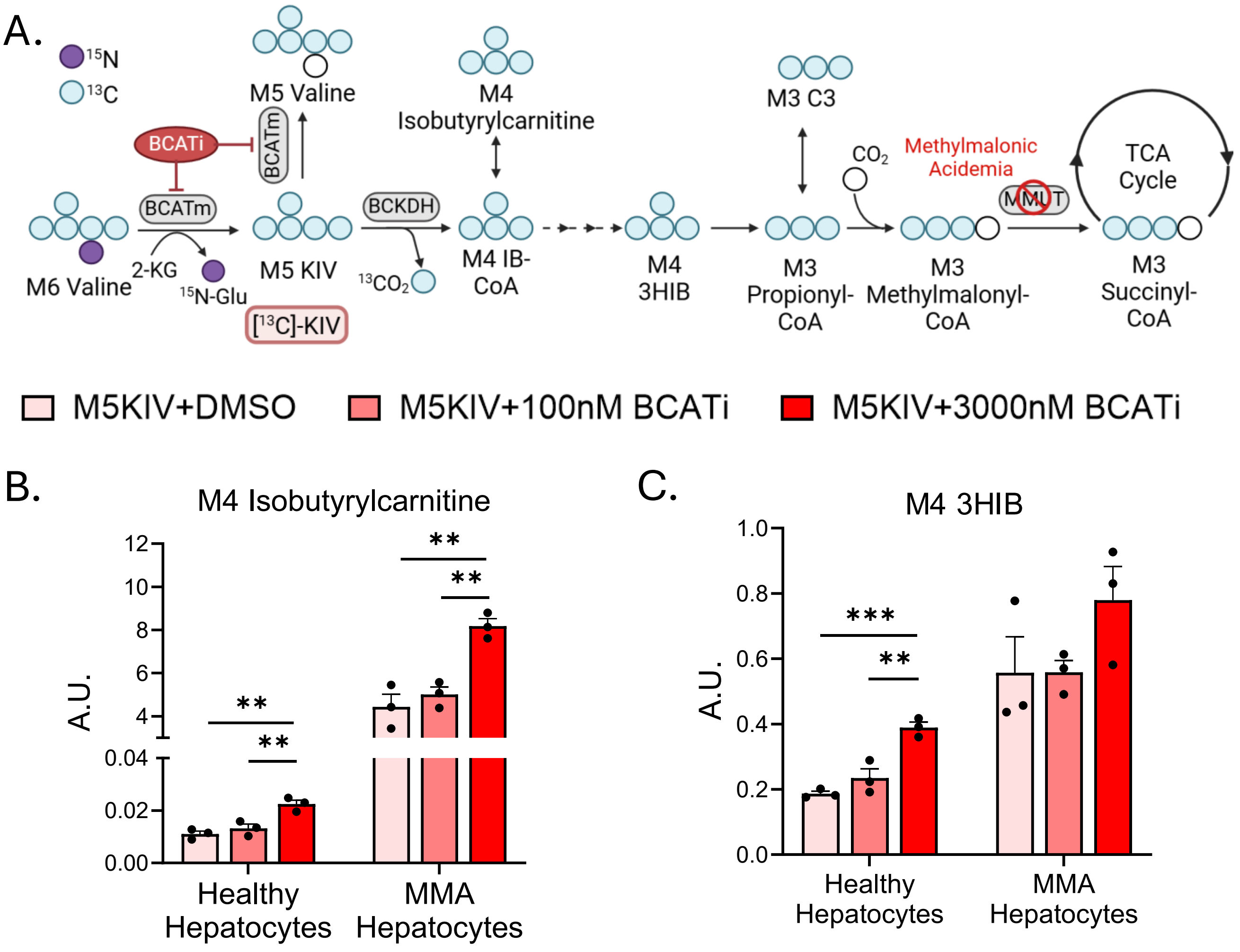

Supplemental Figure 2. Ketoisovalerate (KIV) flux analyses in primary hepatocyte cultures with and without BCATi exposure. Schematic of isotope tracing with labeled [$^{13}C_5$]-KIV**. B.** Quantification of M4 isobutyryl carnitine in healthy and MMA hepatocytes supplemented with 800 µM [$^{13}C_5$]-KIV. **C.** Quantification of M4 3HIB in healthy and MMA hepatocytes supplemented with 800 µM [$^{13}C_5$]-KIV. Data expressed as arbitrary units (A.U.) representing peak areas for a given metabolite relative to peak area of an added norvaline standard for 3HIB and M9 carnitine internal standard for M4 isobutyrylcarnitine. All results are mean + s.e.m. and analyzed by two-way ANOVA. A.U. stands for arbitrary units. "*"p ≤ 0.05, "**" p ≤ 0.01, "***" p ≤ 0.001.

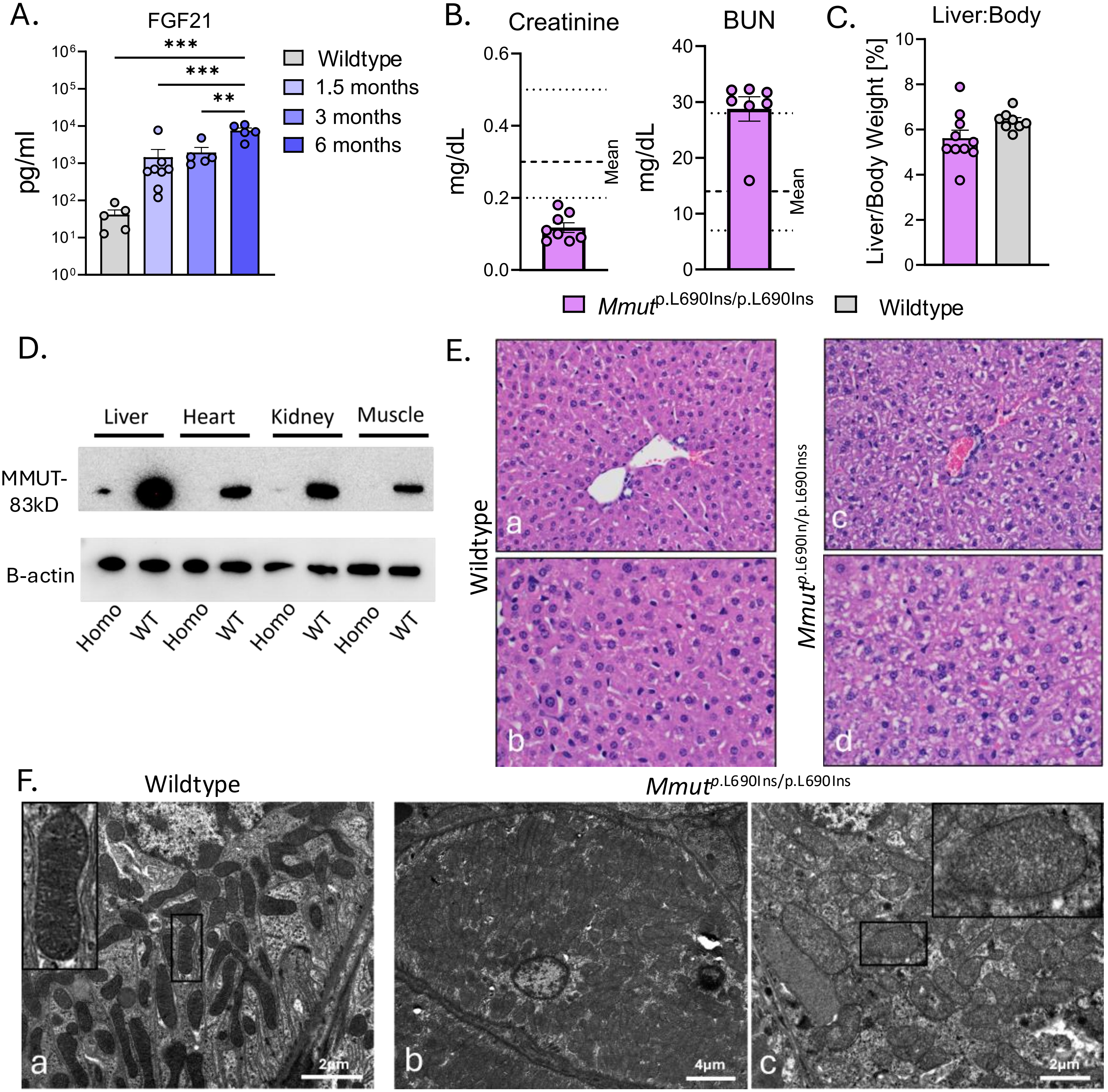

Supplemental Figure 3. A. Characterization of a novel MMA mouse model - the *Mmut*$^{p.L690Ins/p.L690Ins}$ mouse. Plasma FGF21 concentrations in *Mmut*$^{p.L690Ins/p.L690Ins}$ mice of different ages. **B.** Plasma creatinine and blood urea nitrogen (BUN) of *Mmut*$^{p.L690Ins/p.L690Ins}$ mice. C57Bl/6 reference range indicated by dotted lines. **C.** Ratio of liver to body weight, expressed as percent of body weight. **D.** Western blot of MMUT protein expression in liver, heart, kidney, and skeletal muscle tissue from wildtype and homozygous *Mmut*$^{p.L690Ins/p.L690Ins}$ mice. **E.** H&E-stained liver samples. Panels (a) and (b) show portal/periportal and lobular areas from wildtype mice with densely eosinophilic hepatocytes. Panels (c) and (d) show corresponding areas from the *Mmut*$^{p.L690Ins/p.L690Ins}$ mice with less eosinophilic hepatocytes with pale or vacuolated cytoplasm. (a, c) 400x (b,d) 600x magnification. **F.** Electron microscopy of renal tubular epithelial cells. (a) Portion of nucleus on the top surrounded by normally distributed mitochondria in a wildtype mouse. Inset shows normal mitochondrial cristae. (b) Off-center nucleus and densely packed mitochondria that obscure major organelles in a Mmutp.L690Ins/p.L690Ins mouse. (c) Mitochondria show pale matrix and disorganized cristae architecture that is further highlighted in the inset. Bars represent mean + s.e.m. and significance evaluated by one-way ANOVA. "*"$p \leq 0.05$, "**" $p \leq 0.01$, "***" $p \leq 0.001$.

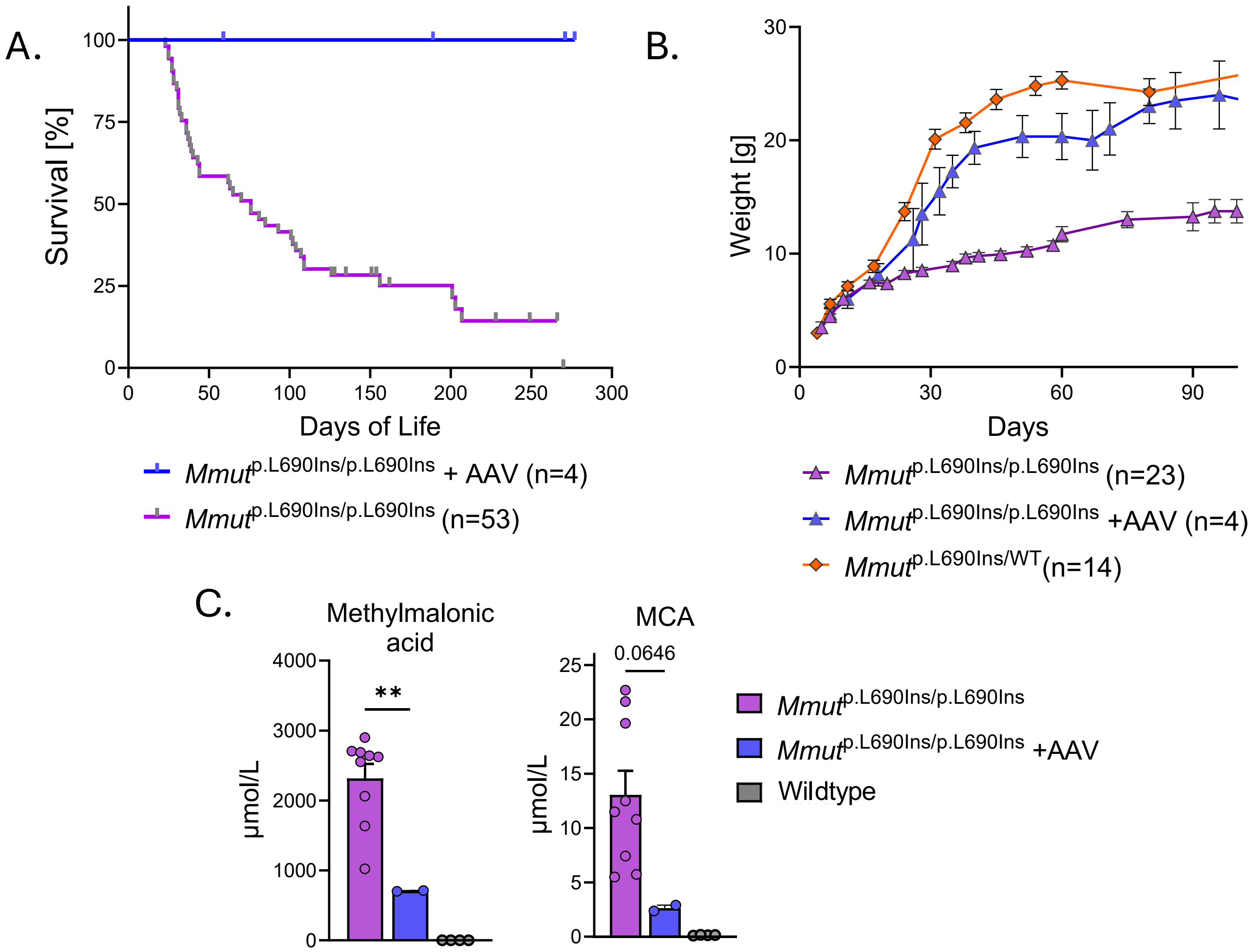

Supplemental Figure 4. Treatment of *Mmut*$^{p.L690Ins/p.L690Ins}$ mice with AAV-hMMUT. A. Survival of *Mmut*$^{p.L690Ins/p.L690Ins}$ mice injected with AAV8-CB7-hMMUT viral gene therapy. **B.** Weight curves of *Mmut*$^{p.L690Ins/p.L690Ins}$ mice. **C.** Quantification of plasma methylmalonic acid and 2-methylcitrate (MCA) in *Mmut*$^{p.L690Ins/p.L690Ins}$ mice injected with AAV8-CB7-hMMUT viral gene therapy. Results are mean + s.e.m. and evaluated by unpaired two-tailed t test. "*" $p \leq 0.05$, "**" $p \leq 0.01$.

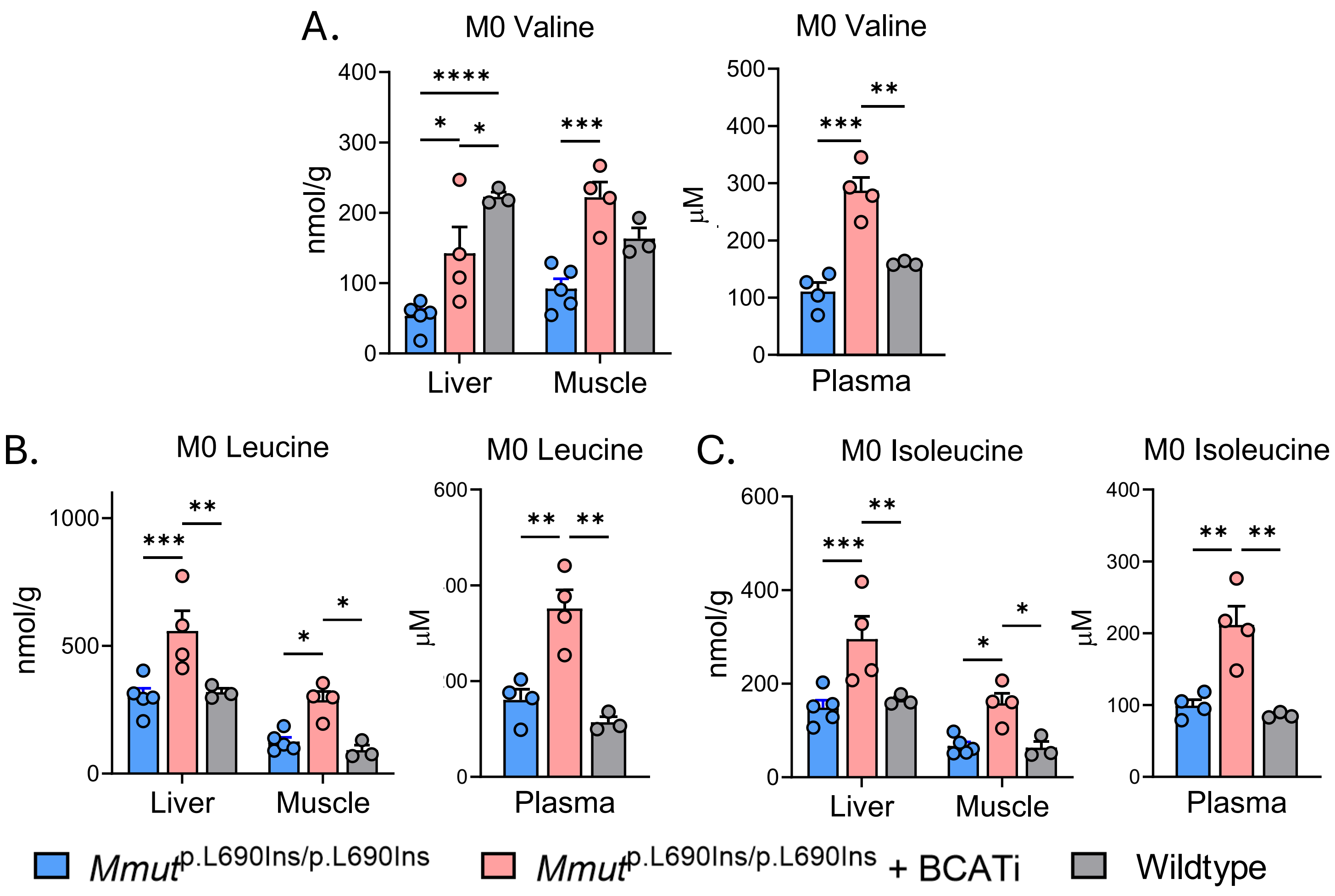

Supplemental Figure 5. Branched chain amino acid levels in different murine tissues in mice with and without BCATi treatment. A-C. Quantification of unlabeled M0 valine **(A.)**, M0 leucine **(B.)**, and M0 isoleucine **(C.)** in liver, skeletal muscle, and plasma, quantified relative to internal standards as described in Methods. Results are mean + s.e.m. and analyzed by one- or two-way ANOVA. A.U. stands for arbitrary units. "*" $p \leq 0.05$, "**" $p \leq 0.01$, "***" $p \leq 0.001$, "****" $p \leq 0.0001$.

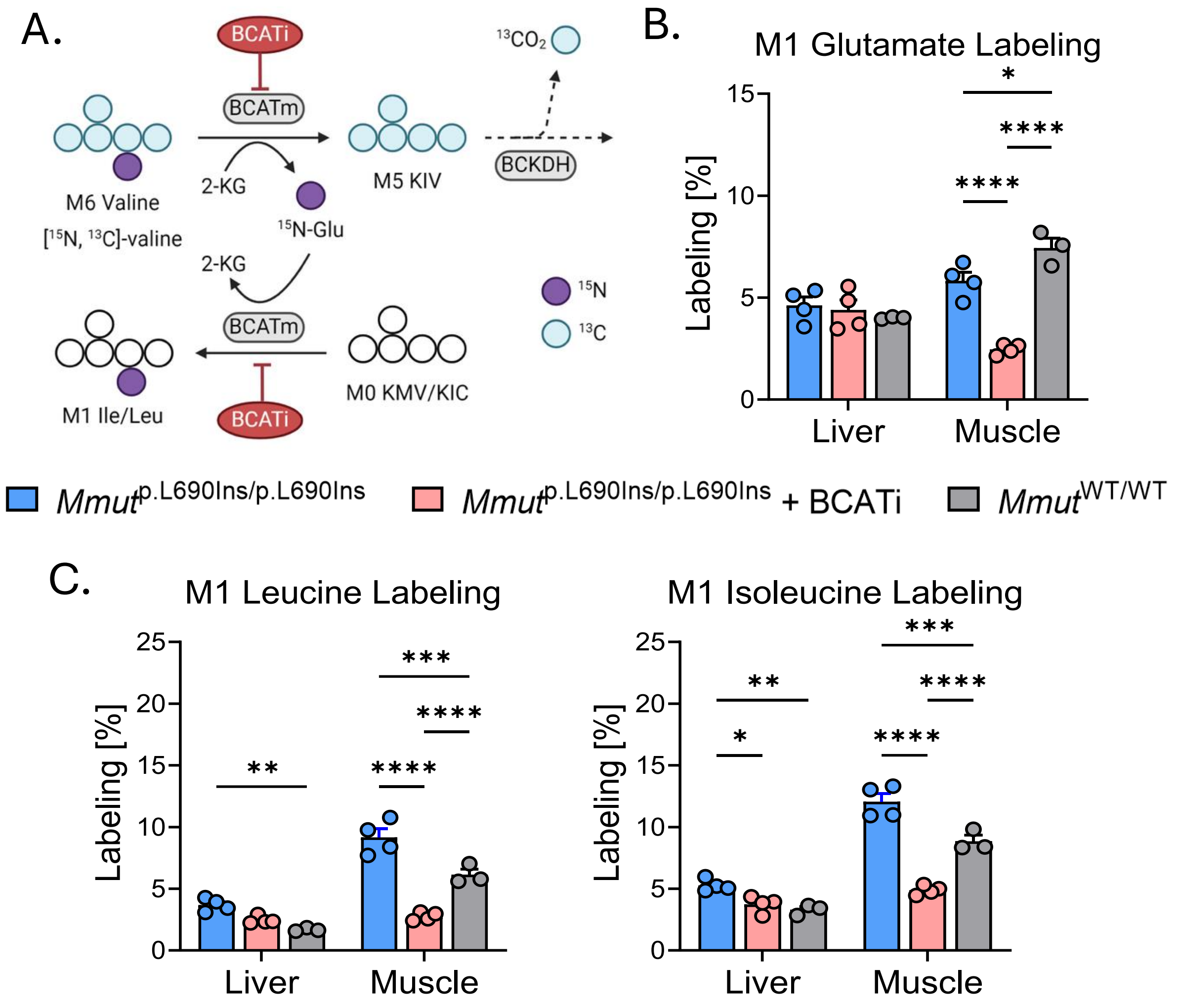

Supplemental Figure 6. Reverse transamination in different murine tissues when treated in mice with and without treatment with BCATi. A. Schematic of two-step reverse labeling of isoleucine and leucine from labeled [^{15}N, $^{13}C_5$]-valine. **B.** Percent labeling of M1 glutamate in liver and skeletal muscle. **C.** Percent labeling of M1 leucine and M1 isoleucine in liver and muscle. All results are mean + s.e.m. and analyzed by two-way ANOVA. “*”$p \leq 0.05$, “**” $p \leq 0.01$, “***” $p \leq 0.001$, “****” $p \leq 0.0001$.
