# Supplementary figures and images for: Dissecting the effect of mitochondrial BCAT inhibition in methylmalonic acidemia
Source: JCI Insight. 2025 Sep 9;10(17):e187758. doi: 10.1172/jci.insight.187758 (PMC12487681; doi:10.1172/jci.insight.187758)

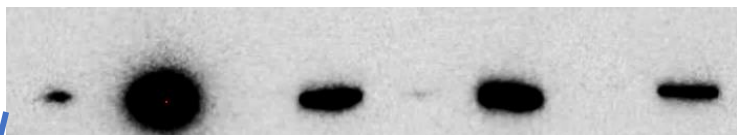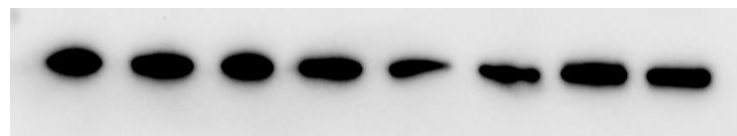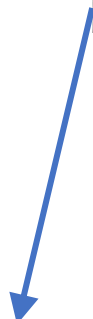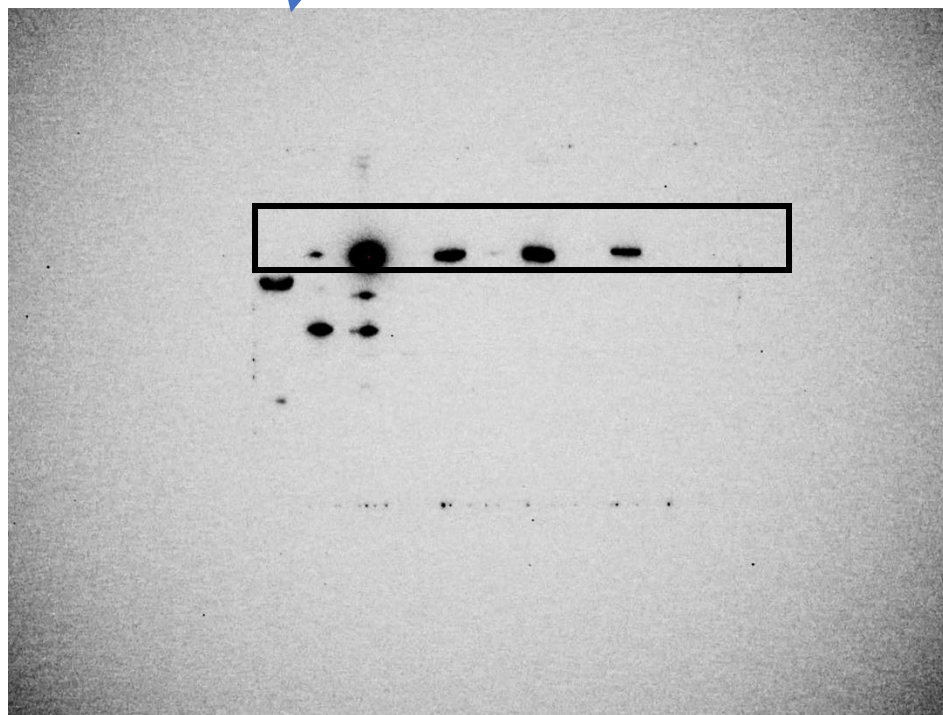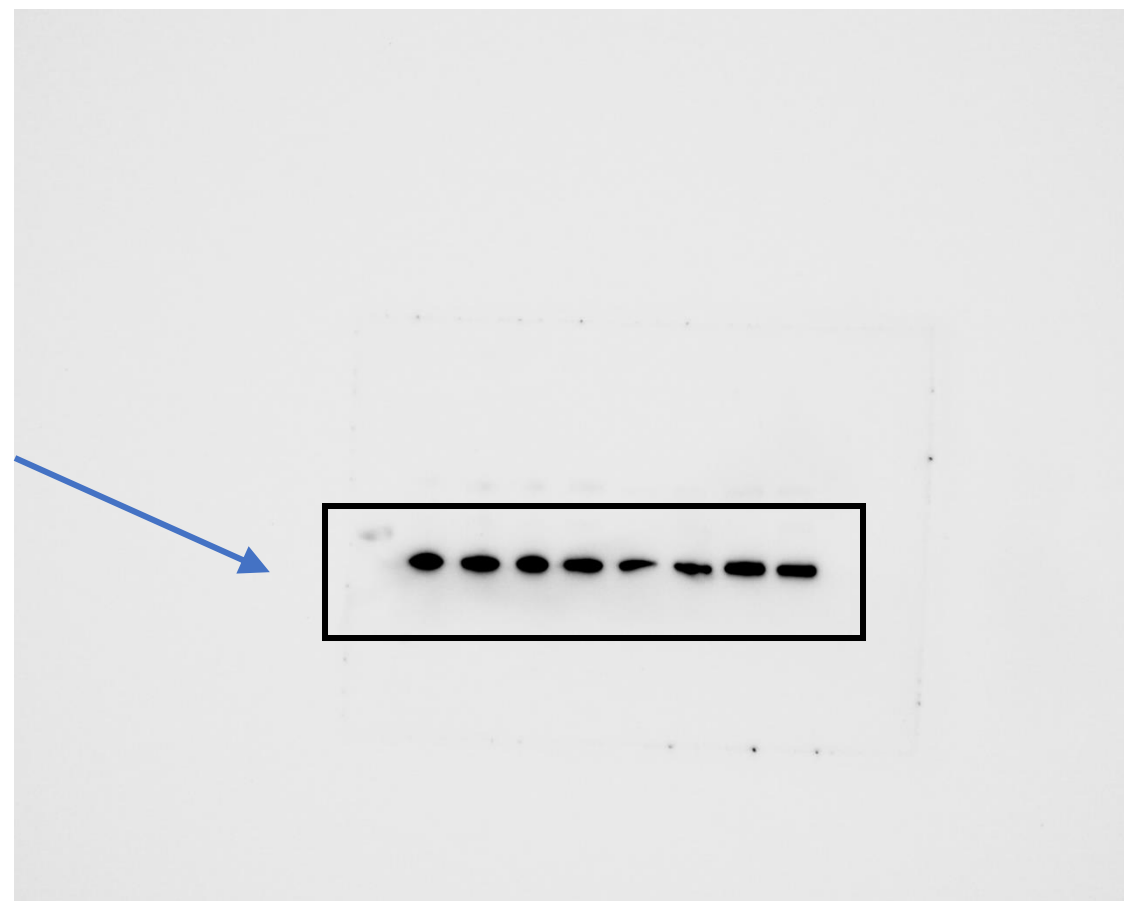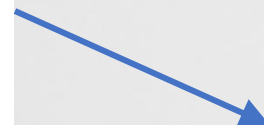

Supplement: Unedited blot and gel images [file jciinsight-10-187758-s048.pdf]
